# SUMOylation-triggered ALIX activation modulates extracellular vesicles circTLCD4-RWDD3 to promote lymphatic metastasis of non-small cell lung cancer

**DOI:** 10.1038/s41392-023-01685-0

**Published:** 2023-11-04

**Authors:** Xiayao Diao, Chao Guo, Hanhao Zheng, Ke Zhao, Yuming Luo, Mingjie An, Yan Lin, Jiancheng Chen, Yuanlong Li, Yuting Li, Xuehan Gao, Jiaqi Zhang, Mengxin Zhou, Wenliang Bai, Lei Liu, Guige Wang, Lanjun Zhang, Xiaotian He, Rusi Zhang, Zhihua Li, Changhao Chen, Shanqing Li

**Affiliations:** 1 Department of Thoracic Surgery, Peking Union Medical College Hospital, Chinese Academy of Medical Sciences and Peking Union Medical College, Beijing, P. R. China; 2 Department of Urology, Sun Yat-sen Memorial Hospital, Sun Yat-sen University, Guangzhou, Guangdong P. R. China; 3 Guangdong Provincial Key Laboratory of Malignant Tumor Epigenetics and Gene Regulation, Sun Yat-sen Memorial Hospital, State Key Laboratory of Oncology in South China, Guangzhou, Guangdong P. R. China; 4 Department of General Surgery, Guangdong Provincial People’s Hospital, Guangdong Academy of Medical Sciences, Guangzhou, Guangdong P. R. China; 5 Cancer Center, Renmin Hospital of Wuhan University, Wuhan, Hubei P. R. China; 6 Department of Thoracic Surgery, Sun Yat-sen University Cancer Center, Sun Yat-sen University, Guangzhou, Guangdong P. R. China; 7 Department of Medical Oncology, Sun Yat-sen Memorial Hospital, Sun Yat-sen University, Guangzhou, Guangdong P. R. China

**Keywords:** Lung cancer, Metastasis

## Abstract

Lymph node (LN) metastasis is one of the predominant metastatic routes of non-small cell lung cancer (NSCLC) and is considered as a leading cause for the unsatisfactory prognosis of patients. Although lymphangiogenesis is well-recognized as a crucial process in mediating LN metastasis, the regulatory mechanism involving lymphangiogenesis and LN metastasis in NSCLC remains unclear. In this study, we employed high-throughput sequencing to identify a novel circular RNA (circRNA), circTLCD4-RWDD3, which was significantly upregulated in extracellular vesicles (EVs) from LN metastatic NSCLC and was positively associated with deteriorated OS and DFS of patients with NSCLC from multicenter clinical cohort. Downregulating the expression of EV-packaged circTLCD4-RWDD3 inhibited lymphangiogenesis and LN metastasis of NSCLC both in vitro and in vivo. Mechanically, circTLCD4-RWDD3 physically interacted with hnRNPA2B1 and mediated the SUMO2 modification at K108 residue of hnRNPA2B1 by upregulating UBC9. Subsequently, circTLCD4-RWDD3-induced SUMOylated hnRNPA2B1 was recognized by the SUMO interaction motif (SIM) of ALIX and activated ALIX to recruit ESCRT-III, thereby facilitating the sorting of circTLCD4-RWDD3 into NSCLC cell-derived EVs. Moreover, EV-packaged circTLCD4-RWDD3 was internalized by lymphatic endothelial cells to activate the transcription of *PROX1*, resulting in the lymphangiogenesis and LN metastasis of NSCLC. Importantly, blocking EV-mediated transmission of circTLCD4-RWDD3 via mutating SIM in ALIX or K108 residue of hnRNPA2B1 inhibited the lymphangiogenesis and LN metastasis of NSCLC in vivo. Our findings reveal a precise mechanism underlying SUMOylated hnRNPA2B1-induced EV packaging of circTLCD4-RWDD3 in facilitating LN metastasis of NSCLC, suggesting that EV-packaged circTLCD4-RWDD3 could be a potential therapeutic target against LN metastatic NSCLC.

## Introduction

Lung cancer ranks as the leading cause of cancer-related deaths worldwide and is the second most frequently diagnosed malignancy globally.^1^ Approximately 85% of lung cancers are non-small cell lung cancers (NSCLC).^2^ Lymph node (LN) metastasis represents a primary route of metastasis in NSCLC and serves as an essential indicator of the patient’s prognosis, with a deteriorated 5-year survival rate of patients from 75% to 20%.^3^ Moreover, the LN status of NSCLC is a pivotal factor in determining the accurate stage of patients and the optimal therapeutic modality.^4^ Although the crucial role of LN metastasis has been well established in NSCLC, the limited understanding of the associated regulatory mechanism greatly impedes the development of effective targeted therapies against LN metastatic NSCLC.

Lymphangiogenesis refers to the sprouting of lymphatic vessels from preexisting lymphatic networks, which represents as a hallmark of metastatic potential and occurs as central and rate-limiting process in tumor LN metastasis by facilitating tumor cell dissemination to the lymphatic system.^5,6^ Previous study has demonstrated that the uncontrolled lymphangiogenesis in tumor tissues is a crucial predictor for the LN metastasis of NSCLC.^7^ The interaction between tumor cells and lymphatic vessels via mutual transmission of multiple signaling molecules is the common manner in stimulating tumor lymphangiogenesis, among which extracellular vesicles (EVs), the endogenous nano-sized membranous particles that transport molecular cargos to mediate intercellular communication and facilitate tumor lymphangiogenesis are under extensive exploration.^8,9^ Targeting tumor cell-derived EVs to block the biological cargo transmission between tumor cells and lymphatic endothelial cells exhibited a great effect in inhibiting tumor lymphangiogenesis and LN metastasis.^10,11^ Nevertheless, the precise biological role and underlying mechanisms of NSCLC-derived EVs in lymphangiogenesis and LN metastasis of NSCLC remain largely unknown and require further elucidation.

Heterogeneous nuclear ribonucleoprotein A2B1 (hnRNPA2B1), a ubiquitously expressed RNA-binding protein, participates in multiple aspects of nucleic acid metabolism by recognizing and binding to particular nucleic acid motif, thus regulating tumor metastasis.^12,13^ Previous study indicated that hnRNPA2B1 was mainly involved in mRNA translation, transport and splicing,^14^ while post-translational modifications (PTMs) reported on hnRNPA2B1 reveal the heterogeneity of hnRNPA2B1-mediated cellular functions.^15^ Demethylation of hnRNPA2B1 results in its activation and the subsequent initiation of IFN-α/β production to further response to DNA virus infection.^16^ Moreover, SUMOylated hnRNPA2B1 is known to recognize and bind to replication protein A (RPA), consequently inhibiting RPA accumulation at replication forks during unperturbed DNA replication.^17^ In addition, oxidative stress induces O-GlcNAcylation of hnRNPA2B1, resulting in the alteration of miRNA repertoire loading into EVs.^18^ These observations reveal that the functional diversity and RNA-recognition specificity of hnRNPA2B1 are mediated by its flexible protein conformations and PTMs. Nonetheless, little is known about whether and how hnRNPA2B1 might be regulated by PTMs to further trigger the LN metastasis in NSCLC.

In the present study, a significantly upregulated circRNA (has_circ_0000095), termed circTLCD4-RWDD3, was identified in NSCLC using high-throughput sequencing, and the overexpression of circTLCD4-RWDD3 in LN metastatic NSCLC was verified in a multicenter cohort of 312 NSCLC cases. We demonstrated that circTLCD4-RWDD3 was enriched in NSCLC cell-derived EVs and promoted the tube formation and migration of human lymphatic endothelial cells (HLECs) in vitro and lymphangiogenesis and LN metastasis in vivo. Mechanically, circTLCD4-RWDD3 transcriptionally upregulated ubiquitin carrier protein 9 (UBC9) expression to facilitate the SUMO2 modification on the K108 residue of hnRNPA2B1, which was then recognized by the SUMO interaction motif (SIM) sequence in ALG-2-Interacting Protein X (ALIX) to activate ALIX and further package circTLCD4-RWDD3 into EVs by recruiting endosomal sorting complexes required for transport (ESCRT)-III. Then, EV-packaged circTLCD4-RWDD3 was transmitted to HLECs and activated prospero homeobox 1 (PROX1) transcription to induce lymphangiogenesis and LN metastasis. These findings uncover a novel mechanism of loading circTLCD4-RWDD3 into NSCLC cell-derived EVs to promote lymphangiogenesis and highlight that EV-packaged circTLCD4-RWDD3 might be a therapeutic target in NSCLC lymphatic metastasis.

## Results

### EV-packaged circTLCD4-RWDD3 correlates with LN metastasis in NSCLC

**Fig. 1:**
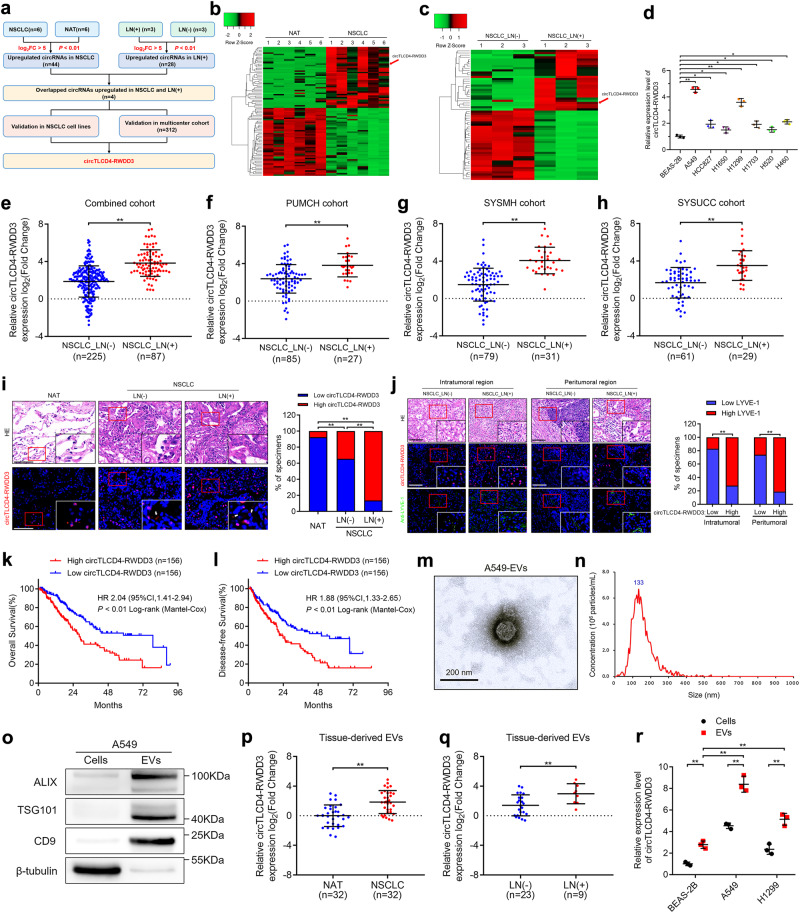
EV-packaged circTLCD4-RWDD3 positively correlates with LN metastasis of NSCLC. **a** Flowchart of steps to identify circRNAs upregulated in NSCLC relative to NATs and upregulated in LN-positive NSCLC relative to LN-negative cancer. **b**, **c** Heatmap of circRNAs differentially expressed in NSCLC tissues and NATs and in NSCLC tissues with or without LN metastasis. **d** qRT-PCR analysis of circTLCD4-RWDD3 expression in NSCLC cell lines and human bronchial epithelial cell line. **e–h** qRT-PCR analysis of circTLCD4-RWDD3 expression in LN-positive and LN-negative NSCLC tissues from PUMCH, SYSMH, SYSUCC, and combined cohort, respectively. **i** Representative FISH images and percentages of circTLCD4-RWDD3 expression in LN-positive or LN-negative NSCLC tissues and NATs. White arrows indicated the extracellular expression of circTLCD4-RWDD3. Scale bars, 50 µm. **j** Representative images and percentages of circTLCD4-RWDD3 expression and LYVE-1-indicated lymphatic vessels in NSCLC tissues. Scale bars, 50 µm. **k**, **l** Kaplan–Meier curves for OS (**k**) and DFS (**l**) of NSCLC patients with low versus high circTLCD4-RWDD3 expression. The cutoff value is the median. **m**, **n** TEM (**m**) and NTA (**n**) identified the characteristics of A549 cell-derived EVs. Scale bar, 200 nm. **o** Western blotting analysis of EV markers in cell lysates or A549 cell-derived EVs. **p** qRT-PCR analysis of circTLCD4-RWDD3 expression in EVs derived from NSCLC tissues and paired NATs. **q** qRT-PCR analysis of circTLCD4-RWDD3 expression in EVs derived from LN-positive and LN-negative NSCLC tissues. **r** qRT-PCR analysis of circTLCD4-RWDD3 expression in NSCLC cell lines and human bronchial epithelial cell line and in their corresponding EVs. The statistical difference was assessed through nonparametric Mann–Whitney *U* test in (**e–h**, **p**, and **q**); and Chi-square test in (**i**, **j**); and one-way ANOVA followed by Dunnett tests in (**d**, **r**); and unpaired Student’s *t*-test in (**r**). Error bars show the SD from three independent experiments. *P < 0.05; **P < 0.01

To characterize the critical circRNAs in promoting LN metastasis in NSCLC, the high-throughput sequencing was performed on 6 pairs of NSCLC tissues and their corresponding normal adjacent tissues (NATs) (GSE235634) (Fig. 1a). The results exhibited 44 circRNAs that were upregulated and 49 that were downregulated in NSCLC tissues compared to NATs (Fig. 1b). Subsequently, we identified 28 circRNAs that were upregulated and 32 that were downregulated in LN-positive NSCLC tissues when compared to LN-negative counterparts (Fig. 1c). Four circRNAs exhibited overlapping upregulation in both NSCLC and LN-positive cancer samples (Fig. 1a). These four circRNAs were expressed at higher levels in various NSCLC cell lines than in normal bronchial epithelial cells (Fig. 1d, Supplementary Fig. 1a–c).

We validated these findings in a three-center cohort of 312 NSCLC patients, in which circTLCD4-RWDD3 (has_circ_0000095) emerged as the most significantly overexpressed circRNA in NSCLC tissues relative to NATs and in LN-positive compared to LN-negative NSCLC (Fig. 1e–h, Supplementary Fig. 1d–m, Supplementary Table 1). This differential expression was further confirmed using fluorescence in situ hybridization of NSCLC tissues, which barely detected the circTLCD4-RWDD3 in NATs (Fig. 1i). Moreover, intratumoral and peritumoral areas of NSCLC tissues with high circTLCD4-RWDD3 expression also showed high density of lymphatic vessel endothelial hyaluronan receptor 1 (LYVE-1)-positive microlymphatic vessels (Fig. 1j).

Upon stratifying NSCLC patients based on their circTLCD4-RWDD3 expression levels relative to the median, we observed a significant correlation between higher circTLCD4-RWDD3 expression and worse overall survival (OS) and disease-free survival (DFS) of patients (Fig. 1k, l). Univariate and multivariate Cox analyses identified circTLCD4-RWDD3 as an independent predictor of OS and DFS of patients with NSCLC (Supplementary Table 2, 3).

The analysis of the 410-nt sequence of circTLCD4-RWDD3 suggested that it is formed by back-splicing of *TLCD4-RWDD3* transcripts from exon 2 to 5 (Supplementary Fig. 1n), which was validated by Sanger sequencing (Supplementary Fig. 1o). The divergent primers could amplify circTLCD4-RWDD3 only in cDNA but not in genomic DNA from NSCLC cells (Supplementary Fig. 1p). Using oligo-dT primers instead of random primers for reverse transcription, the expression of circTLCD4-RWDD3 was significantly decreased, indicating the absence of poly (A) tailed structure in circTLCD4-RWDD3 (Supplementary Fig. 1q). Moreover, treatment with RNase R on total RNA led to a substantial reduction in *TLCD4-RWDD3* mRNA levels, while circTLCD4-RWDD3 unaffected, consistent with its closed circular structure (Supplementary Fig. 1r). Treating NSCLC cell lines with actinomycin D led to faster decay of *TLCD4-RWDD3* mRNA than circTLCD4-RWDD3 (Supplementary Fig. 1s, t), confirming its stability.

Interestingly, FISH assays with probes targeting circTLCD4-RWDD3 revealed the expression of circTLCD4-RWDD3 in the extracellular regions of NSCLC tissues, and higher extracellular circTLCD4-RWDD3 expression in LN-positive NSCLC tissues compared with LN-negative NSCLC tissues (Supplementary Fig. 1u), suggesting that circTLCD4-RWDD3 might involve in NSCLC LN metastasis in its extracellular form. It has been reported that EVs are crucial nano-sized mediators of cellular crosstalk by packaging various biomolecules and crossing the extracellular matrix into lymphatic organs.^19^ Thus, we investigated circTLCD4-RWDD3 expression in EVs from NSCLC tissues and in the culture medium (CM) of NSCLC cell lines. Transmission electron microscopy (TEM) and nanoparticle tracking analysis (NTA) were utilized to identify the cup-shaped morphology and 30–150 nm size distribution of isolated vesicles (Fig. 1m, n, Supplementary Fig. 1v, w). Moreover, the isolated vesicles contained well-established EV markers, including ALIX, TSG101, and CD9, confirming the isolated vesicles are EVs (Fig. 1o). The levels of circTLCD4-RWDD3 were significantly higher in EVs derived from NSCLC tissues than in those derived from paired NATs (Fig. 1p), and in EVs from LN-positive NSCLC tissues than in those from LN-negative tissues (Fig. 1q).

Furthermore, circTLCD4-RWDD3 expression was notably enriched in EVs derived from both NSCLC cells and BEAS-2B cells, while EVs derived from A549 and H1299 cells displayed higher expression levels of circTLCD4-RWDD3 compared to other cell lines (Fig. 1r, Supplementary Fig. 2a). In addition, circTLCD4-RWDD3 expression in A549 and H1299 cell-secreted EVs was dramatically upregulated or downregulated by circTLCD4-RWDD3 plasmid transfection or knocking down in corresponding cell lines (Supplementary Fig. 2b–e), indicating that alteration of cellular circTLCD4-RWDD3 expression markedly affects EV-packaged circTLCD4-RWDD3 expression. Taken together, these findings illustrate that EV-packaged circTLCD4-RWDD3 positive correlates with LN metastasis in NSCLC.

### EV-packaged circTLCD4-RWDD3 promotes lymphangiogenesis of NSCLC in vitro

**Fig. 2:**
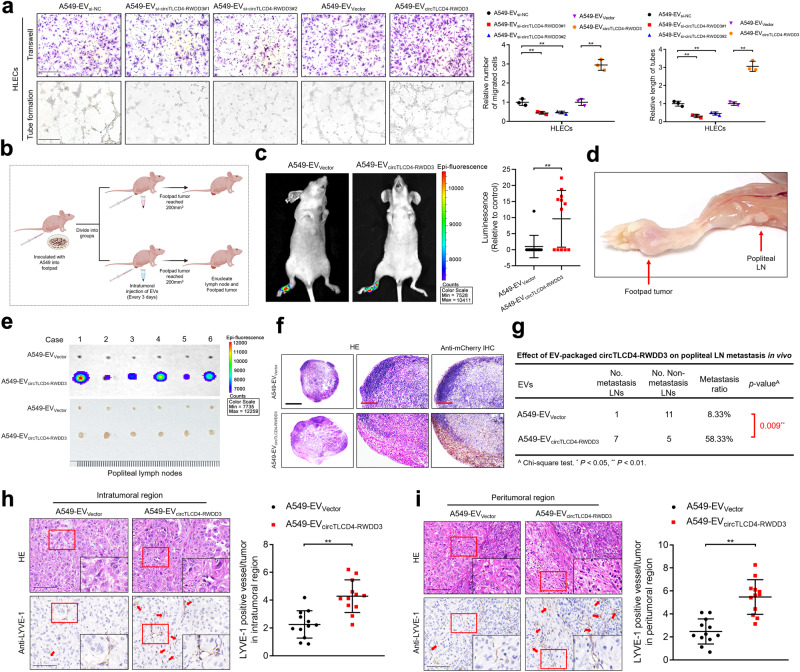
EV-packaged circTLCD4-RWDD3 promotes lymphangiogenesis and LN metastasis in NSCLC in vitro and in vivo. **a** Representative images and quantification of the tube formation and Transwell migration of HLECs treated with circTLCD4-RWDD3-downregulating or -upregulating A549 cell-derived EVs. Scale bars, 100 µm. **b** Schematic diagram of the construction of a nude mice popliteal LN metastasis model. Schematic was created with BioRender (www.biorender.com). **c** Representative images and quantification of bioluminescence of the popliteal LN metastasis in nude mice (*n* = 12 per group). **d** Representative image of the footpad primary tumor and popliteal metastatic LN in a nude mouse. **e** Representative bioluminescence image of excised popliteal LNs from the nude mice (*n* = 12 per group). **f** Representative anti-mCherry IHC images of nude mice popliteal LNs (*n* = 12 per group). Scale bars, 500 µm (black); scale bars, 100 µm (red). **g** The percentage of metastatic popliteal LN in the nude mice (*n* = 12 per group). **h**, **i** Representative IHC images and percentages of LYVE-1-indicated lymphatic vessel density in intratumoral (**h**) and peritumoral region (**i**) of footpad primary tumor tissues. Scale bars, 50 µm. The statistical difference was assessed with one-way ANOVA followed by Dunnett tests in (**a**); and unpaired Student’s *t*-test in (**a**, **c**, **h**, and **i**); and Chi-square test in (**g**). Error bars show the SD from three independent experiments. *P < 0.05; **P < 0.01

Given the recognized pivotal role of lymphangiogenesis in mediating tumor LN metastasis,^6^ we investigated the effect of EV-packaged circTLCD4-RWDD3 on lymphangiogenesis of NSCLC in vitro. EVs from A549 and H1299 cells that were previously proved to exhibit a high propensity for LN metastasis promoted migration and tube formation by HLECs to a significantly greater extent when compared to EVs from other NSCLC cell lines or BEAS-2B normal lung epithelial cells (Supplementary Fig. 2f).^20^ This observation was consistent with the higher levels of circTLCD4-RWDD3 in the vesicles from A549 and H1299 cells (Fig. 1r). Moreover, EVs derived from A549 and H1299 cells with circTLCD4-RWDD3 knockdown significantly impaired HLECs migration and tube formation compared with the control group, while the migration and tube formation of HLECs were significantly enhanced by EVs derived from A549 and H1299 cells with upregulated circTLCD4-RWDD3 (Fig. 2a, Supplementary Fig. 2g). Additionally, we successfully established circTLCD4-RWDD3-knockout (circTLCD4-RWDD3^KO^) NSCLC cell lines by transfecting paired gRNAs targeting the boundaries of the Alu sequence in the intron upstream of TLCD4-RWDD3 exon 2 (Supplementary Fig. 2h, i), thereby deleting the critical Alu element and demonstrated that EVs derived from circTLCD4-RWDD3^KO^ A549 and H1299 cells markedly suppressed the tube formation and migration of HLECs compared with the control group (Supplementary Fig. 2j–m). Collectively, these results demonstrate that EV-packaged circTLCD4-RWDD3 secreted by NSCLC cells facilitates the tube formation and migration of HLECs to induce lymphangiogenesis of NSCLC in vitro.

### EV-packaged circTLCD4-RWDD3 facilitates LN metastasis of NSCLC in vivo

We examined the potential pro-metastatic effects of circTLCD4-RWDD3 in a mouse model of NSCLC metastasis to popliteal LNs.^10,21^ In this model, mCherry-labeled A549 cells were inoculated into the footpads of nude mice, followed by injections of EVs from A549 cells expressing either normal or elevated levels of circTLCD4-RWDD3 (A549-EV_Vector_ or A549-EV_circTLCD4-RWDD3_) into the resulting primary tumors every three days. The primary tumors and popliteal LNs were resected and subjected to immunohistochemistry (IHC) analysis when the tumor size reached about 200 mm^3^ (Fig. 2b). As demonstrated by in vivo imaging system (*IVIS*), A549-EV_circTLCD4-RWDD3_ facilitated the metastasis of NSCLC cells to the popliteal LNs of nude mice compared with A549-EV_Vector_ (Fig. 2c). Accordingly, larger popliteal LN volume and increased LN metastasis rate were showed in the group treated with EVs from circTLCD4-RWDD3-overexpressing NSCLC cells than in the control group (Fig. 2d–g). To further evaluate the effect of EV-packaged circTLCD4-RWDD3 on lymphangiogenesis in vivo, we performed IHC staining to reveal that LYVE-1-labeled microlymphatic vessel density (MLD) was significantly increased in both the intratumoral and peritumoral regions of the footpad primary tumor tissues from the group treated with EVs from circTLCD4-RWDD3-overexpressing NSCLC cells than in the control group (Fig. 2h, i), confirming that EV-packaged circTLCD4-RWDD3 induces NSCLC lymphangiogenesis. Collectively, these results suggest that EV-packaged circTLCD4-RWDD3 enhances the lymphangiogenesis and LN metastasis of NSCLC in vivo.

### circTLCD4-RWDD3 binds directly to hnRNPA2B1 in NSCLC cells

**Fig. 3:**
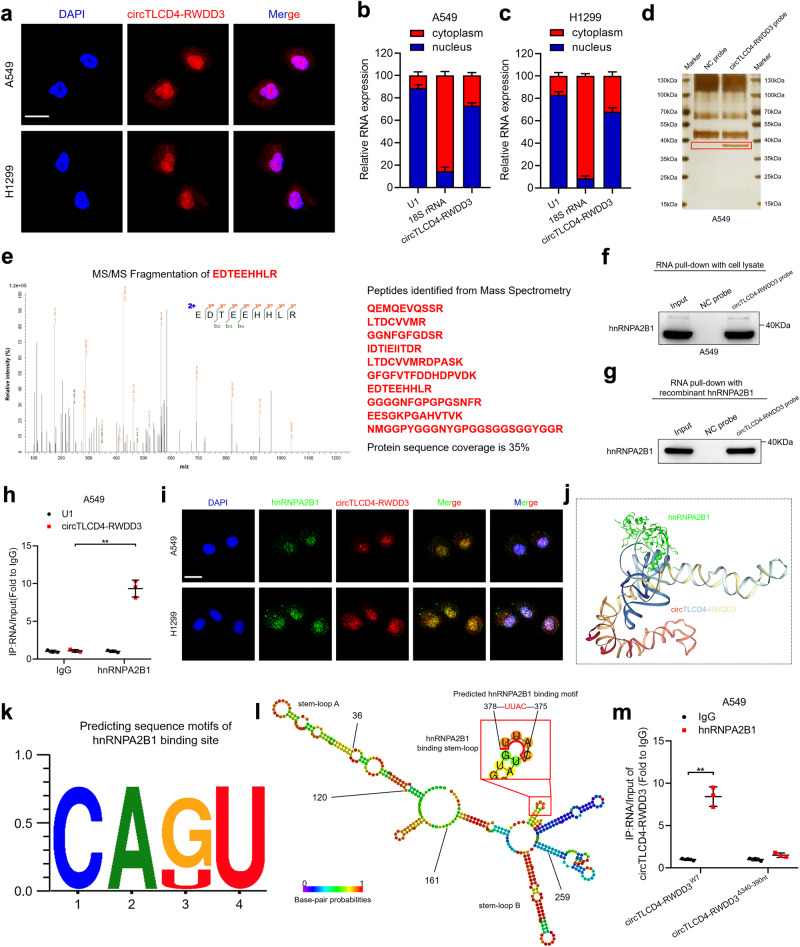
circTLCD4-RWDD3 directly interacts with hnRNPA2B1 in NSCLC cells. **a–c** Detection of intracellular localization of circTLCD4-RWDD3 in NSCLC cells using FISH assays (**a**) and subcellular fraction assays (**b**, **c**). Scale bars, 5 µm. **d** Silver staining image of RNA pull-down assay with circTLCD4-RWDD3 and control probes in A549 cells. **e** Mass spectrometry analysis of circTLCD4-RWDD3-binding proteins after RNA pull-down assay. **f**, **g** Western blotting analysis after RNA pull-down assay to investigate the interaction between circTLCD4-RWDD3 and hnRNPA2B1. **h** RIP assays with anti-hnRNPA2B1 revealing the enrichment of circTLCD4-RWDD3 by hnRNPA2B1 in A549 cells. **i** Detection of intracellular co-localization of circTLCD4-RWDD3 and hnRNPA2B1 in NSCLC cells. Scale bars, 5 µm. **j** 3D schematic diagram predicting the interaction between circTLCD4-RWDD3 and hnRNPA2B1. **k** hnRNPA2B1-binding motif predicted by RBPmap. **l** The stem-loop structure of hnRNPA2B1-binding motifs in circTLCD4-RWDD3. **m** RIP assays after mutating of the 340–390 nt region of circTLCD4-RWDD3 in A549 cells. The statistical difference was assessed by unpaired Student’s *t*-test in (**h**, **m**). Error bars show the SD from three independent experiments. *P < 0.05; **P < 0.01

Given the ability of circTLCD4-RWDD3 in EVs to promote LN metastasis of NSCLC, we embarked on an exploration of its intracellular functions in parental cells before being packaged into EVs. Since the biological function of circRNA depends on its subcellular location,^22^ we evaluated the specific location of circTLCD4-RWDD3 within A549 and H1299 cells. Our analysis revealed that a substantial portion of circTLCD4-RWDD3 was detected in the nucleus, with a proportion also present in the cytoplasm (Fig. 3a–c). Previous studies have proposed that nuclear circRNAs mainly exert their biological function through interacting with RNA-binding proteins.^23,24^ Thus, we employed biotinylated probes to precipitate circTLCD4-RWDD3 from cells and searched for the potential protein interactors (Supplementary Fig. 3a, b). The precipitate of circTLCD4-RWDD3 probes contained an obvious band with apparent molecular weight of 35–40 kDa compared with NC probes (Fig. 3d, Supplementary Fig. 3c), which was then identified by mass spectrometry (MS) analysis to search the potential proteins interacted with circTLCD4-RWDD3 (Supplementary Table 4). Among the proteins identified by MS analysis, hnRNPA2B1 was confirmed as the most abundant binding protein of circTLCD4-RWDD3 via western blotting analysis after RNA pull-down assays with A549 lysates or with a simple mixture of circTLCD4-RWDD3 and recombinant hnRNPA2B1 (Fig. 3e–g, Supplementary Fig. 3d–g). Consistently, using anti-hnRNPA2B1 antibody to precipitate RNA from A549 cells pulled down significantly more circTLCD4-RWDD3 but not linear TLCD4-RWDD3 than using IgG (Fig. 3h, Supplementary Fig. 3h–j). The distribution of circTLCD4-RWDD3 within A549 and H1299 cells overlapped extensively with that of hnRNPA2B1 (Fig. 3i), indicating that circTLCD4-RWDD3 interacts with hnRNPA2B1 for function in NSCLC. The HDOCK structural alignment tool suggested the formation of a complex between circTLCD4-RWDD3 and hnRNPA2B1 (Fig. 3j), and POSTAR3 sequence analysis predicted that hnRNPA2B1 bound to a stem-loop structure involving nucleotides 340–390 of circTLCD4-RWDD3 through CAUU motif (Fig. 3k–l). Mutating nucleotides 340–390 region or CAUU motif of circTLCD4-RWDD3 markedly impaired the enrichment of circTLCD4-RWDD3 by hnRNPA2B1 (Fig. 3m, Supplementary Fig. 3k–m), suggesting that the nucleotides 340–390 of circTLCD4-RWDD3 are essential for its interaction with hnRNPA2B1. Moreover, we also explored the domain in hnRNPA2B1 that responsible for hnRNPA2B1-circTLCD4-RWDD3 interaction. HnRNPA2B1 comprises two RNA recognition motif (RRM) domains (Supplementary Fig. 3n), among which the RRM2 domain in hnRNPA2B1 was confirmed as the essential domain required for the interaction with circTLCD4-RWDD3 via RIP assays with truncated hnRNPA2B1 (Supplementary Fig. 3o). Collectively, these results demonstrate that circTLCD4-RWDD3 directly interacts with hnRNPA2B1 in NSCLC.

### circTLCD4-RWDD3 upregulates UBC9 to catalyze SUMO2 modification on lysine 108 residue on hnRNPA2B1

**Fig. 4:**
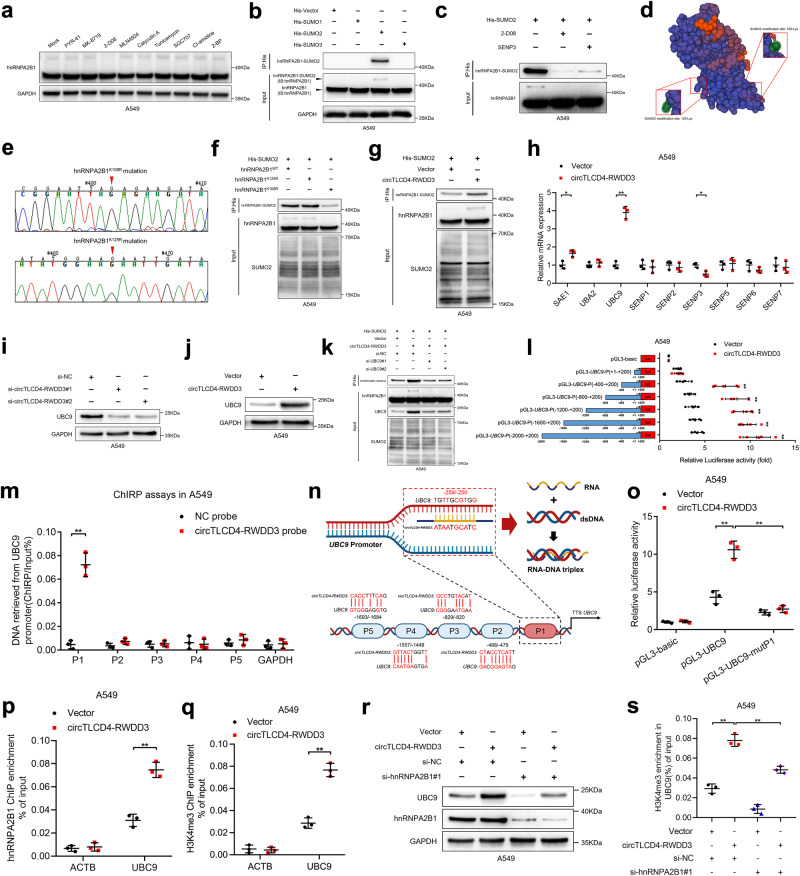
circTLCD4-RWDD3 induces SUMO2 modification at K108 residue of hnRNPA2B1 by upregulating UBC9. **a** Western blotting analysis of hnRNPA2B1 expression in A549 cells treated with various inhibitors of PTM. PYR-41 for ubiquitylation, MK-8719 for O-GlcNAcylation, 2-D08 for SUMOylation, MLN4924 for NEDDylation, Calyculin A for phosphorylation, Tunicamycin for N-linked glycosylation, SGC707 for arginine methylation, CI-amidine for deimination and 2-BP for palmitoylation. **b** Co-IP assay to investigate the SUMOylation type of hnRNPA2B1 in A549 cells. **c** Co-IP assay to assess SUMO2 modification of hnRNPA2B1 after 2-D08 treatment or overexpressing SENP3. **d** Schematic illustration of the SUMOylation sites on hnRNPA2B1 predicted by GPS-SUMO. **e** Sanger sequencing to confirm the hnRNPA2B1^K108R^ and hnRNPA2B1^K125R^ mutations. **f** Western blotting analysis to confirm the SUMO2 modification site on hnRNPA2B1. **g** Western blotting analysis of SUMO2 modification on hnRNPA2B1 in A549 cells with or without circTLCD4-RWDD3 overexpression. **h** qRT-PCR analysis for SUMOylation-related enzyme expression in A549 cells with or without circTLCD4-RWDD3 overexpression. **I**, **j** Western blotting analysis to confirm UBC9 expression after circTLCD4-RWDD3 downregulation (**i**) or overexpression (**j**) in A549 cells. **k** Western blotting analysis to investigate SUMO2 modification on hnRNPA2B1 in circTLCD4-RWDD3-overexpressing A549 cells with or without knocking down UBC9. **l** Transcriptional activity of *UBC9* in circTLCD4-RWDD3-overexpressing A549 cells transfected with truncated *UBC9* promoter luciferase plasmids. **m** ChIRP assays to investigate the circTLCD4-RWDD3-associated chromatin fragments of the *UBC9* promoter in A549 cells. **n** Schematic illustration of the DNA-RNA triplex structure between circTLCD4-RWDD3 and the *UBC9* promoter. Schematic was created with BioRender (www.biorender.com). **o** Luciferase activity detected in circTLCD4-RWDD3-overexpressing A549 cells with or without mutating the circTLCD4-RWDD3-binding site on *UBC9* promoter. **p**, **q** ChIP-qPCR of hnRNPA2B1 (**p**) and H3K4me3 (**q**) enrichment on *UBC9* promoter after upregulating circTLCD4-RWDD3 in A549 cells. (**r**) Western blotting analysis of *UBC9* expression in circTLCD4-RWDD3-overexpressing A549 cells with or without knocking down hnRNPA2B1. (**s**) ChIP-qPCR analysis of H3K4me3 enrichment on *UBC9* promoter in circTLCD4-RWDD3-overexpressing A549 cells with or without knocking down hnRNPA2B1. The statistical difference was assessed by unpaired Student’s *t*-test in (**h**, **l**, **m**, **p**, and **q**); and one-way ANOVA followed by Dunnett tests in (**o**, **s**). Error bars show the SD from three independent experiments. *P < 0.05; **P < 0.01

Interestingly, we noticed that hnRNPA2B1 pulldown by circTLCD4-RWDD3 had an additional band with a higher molecular weight (>40 kDa) than its theoretical molecular weight (Fig. 3f, Supplementary Fig. 3f). It has been proposed that hnRNPA2B1, depending on the cell type and conditions, can be demethylated,^16^ SUMOylated^17^ and O-GlcNAcylated,^18^ and these PTMs allow it to engage in diverse functions.^15^ Therefore, we hypothesized that the >40 kDa band might represent a post-translationally modified form of hnRNPA2B1. To investigate this hypothesis, we treated A549 cells with inhibitors against each type of PTM. Remarkably, only the SUMOylation inhibitor 2-D08 significantly reduced expression of the >40 kDa band (Fig. 4a), suggesting that circTLCD4-RWDD3-bound hnRNPA2B1 is partially SUMOylated in NSCLC. To further corroborate this finding, we stably expressed His-tagged SUMO1, SUMO2, or SUMO3 in A549 cells and performed coimmunoprecipitation (co-IP) assays with anti-His antibody. Notably, hnRNPA2B1 was detected only in the His-SUMO2 overexpressing cells (Fig. 4b). Furthermore, treating with 2-D08 or overexpressing SUMO-specific peptidase 3 (SENP3), a SUMO protease mainly dissociating SUMO2 from substrates,^25^ significantly suppressed the attachment of SUMO2 to hnRNPA2B1 (Fig. 4c), confirming that hnRNPA2B1 is SUMOylated with SUMO2 in NSCLC cells. To further identify the specific SUMOylated residue on hnRNPA2B1, GPS-SUMO, an online predicting tool for SUMOylation, was utilized to predict two potential SUMO2 conjunction residues on hnRNPA2B1, lysine 108 (K108) and lysine 125 (K125) (Fig. 4d). Then, we mutated these potential lysine residues to arginine (R) (hnRNPA2B1^K108R^, hnRNPA2B1^K125R^) and conducted co-IP assays to confirm the SUMOylated residue on hnRNPA2B1 (Fig. 4e). As illustrated in Fig. 4f, hnRNPA2B1^K108R^ rather than hnRNPA2B1^K125R^ markedly attenuated the SUMOylation of hnRNPA2B1, suggesting that K108 is the SUMOylation site of hnRNPA2B1.

Strikingly, overexpressing circTLCD4-RWDD3 significantly enhanced the SUMO2 modification on hnRNA2B1, while knocking down circTLCD4-RWDD3 exhibited the opposite effect (Fig. 4g, Supplementary Fig. 3p), suggesting that circTLCD4-RWDD3 stimulates SUMOylation of hnRNPA2B1. Given that PTMs, including SUMOylation, are typically mediated by enzymatic activity,^26^ we sought to identify the specific SUMOylation-related enzymes regulated by circTLCD4-RWDD3 to illustrate the mechanism of circTLCD4-RWDD3 in regulating SUMOylation of hnRNPA2B1. Among the various SUMOylation-related enzymes examined, we found that the expression of UBC9, a single E2 conjugating enzyme for SUMOylation, was most significantly changed after altering circTLCD4-RWDD3 expression (Fig. 4h, Supplementary Fig. 3q–s), in which UBC9 was strongly downregulated when endogenous circTLCD4-RWDD3 was knocked down and upregulated when circTLCD4-RWDD3 was overexpressed (Fig. 4I, j, Supplementary Fig. 3t, u). Importantly, the increased SUMO2 modification on hnRNPA2B1 induced by circTLCD4-RWDD3 was impaired by downregulating UBC9 expression in NSCLC cells (Fig. 4k). These results suggest that circTLCD4-RWDD3 upregulates UBC9 to catalyze the SUMOylation of hnRNPA2B1 in NSCLC.

To investigate the mechanisms underlying circTLCD4-RWDD3-activated UBC9 upregulation, we conducted luciferase assays involving truncations of the *UBC9* promoter ranging from nucleotide −2000 to nucleotide +200. The results revealed that the transcription activities of nucleotide −400 to 0 region of *UBC9* promoter (referred to as P1) was significantly activated by circTLCD4-RWDD3 overexpression (Fig. 4l, Supplementary Fig. 4a). Chromatin isolation by RNA purification (ChIRP) assays showed that circTLCD4-RWDD3 directly bound with the P1 region of the *UBC9* promoter (Fig. 4m, Supplementary Fig. 4b, c). Through sequence analysis, we predicted that circTLCD4-RWDD3 bound to the region from nucleotides −259 to −250 in the *UBC9* promoter (Fig. 4n). Indeed, mutating this region strongly reduced the ability of circTLCD4-RWDD3 to upregulate the luciferase activity of the *UBC9* promoter (Fig. 4o, Supplementary Fig. 4d). These results suggest that circTLCD4-RWDD3 interacts with the region from nucleotides −259 to −250 in the *UBC9* promoter, forming a DNA-RNA triplex to activate the transcription of *UBC9*.

It has been reported that DNA-RNA triplex, a non-canonical nucleic acid structures, participates in the transcriptional activation through recruiting transcription factors,^27^ among which hnRNPA2B1 has been previously reported to involve in H3K4 trimethylation (H3K4me3) at target genes to further activate their transcription.^13,28^ Therefore, we sought to examine whether circTLCD4-RWDD3 activated *UBC9* transcription by directly interacting with *UBC9* promoter to recruit hnRNPA2B1 and induce the H3K4me3 on *UBC9* promoter. Chromatin immunoprecipitation (ChIP) assays showed that the enrichment of hnRNPA2B1 and H3K4me3 on *UBC9* promoter were significantly increased in circTLCD4-RWDD3-overexpressing NSCLC cells, and knocking down circTLCD4-RWDD3 impeded the enrichment of hnRNPA2B1 and H3K4me3 on *UBC9* promoter (Fig. 4p, q, Supplementary Fig. 4e–n). Knocking down total hnRNPA2B1 instead of mutating the K108 residue of hnRNPA2B1 to impair its SUMOylation reversed the ability of circTLCD4-RWDD3 overexpression to upregulate UBC9 and promote H3K4me3 modification at the *UBC9* promoter (Fig. 4r, s, Supplementary Fig. 4o–s). Moreover, the promoted effect of circTLCD4-RWDD3 in UBC9 expression was attenuated by overexpressing lysine demethylase 5B (KDM5B) to inhibit H3K4me3 (Supplementary Fig. 4t–v).^29^ Taken together, these results suggest that circTLCD4-RWDD3 upregulates UBC9 to catalyze hnRNPA2B1 SUMOylation.

### SUMOylated hnRNPA2B1 sustains ALIX activation via recognizing the SIM sequence on ALIX

**Fig. 5:**
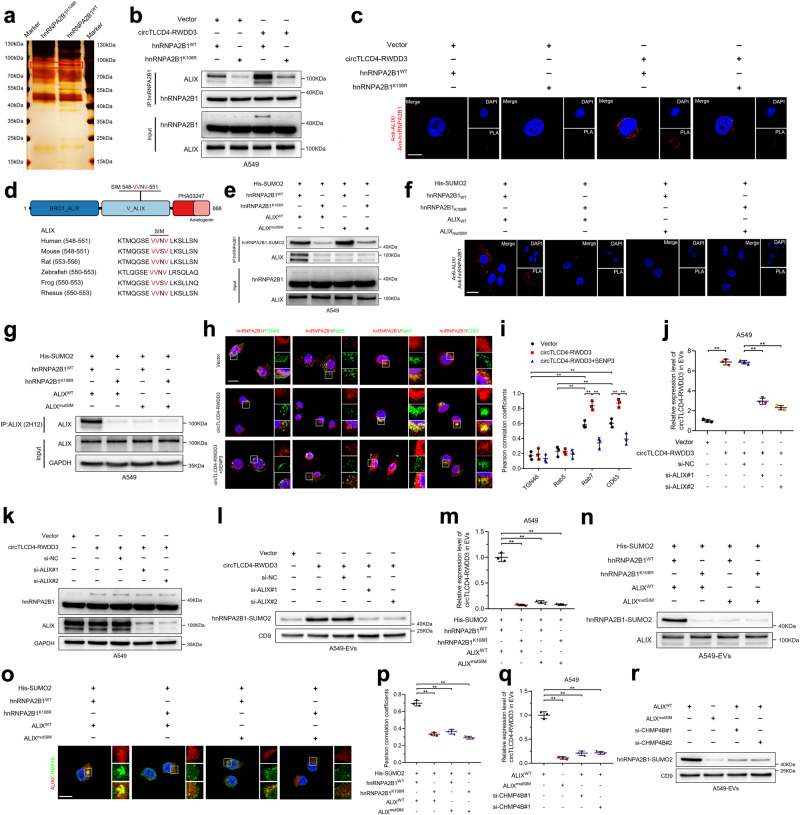
SUMOylated hnRNPA2B1 activates ALIX to trigger circTLCD4-RWDD3 loading into EVs in ESCRT-III-dependent manner. **a** Silver staining after co-IP assay to detect SUMOylated hnRNPA2B1-interacting proteins. **b** Western blotting analysis to assess the interaction between hnRNPA2B1 and ALIX in circTLCD4-RWDD3-overexpressing A549 cells with or without mutating K108 residue of hnRNPA2B1. **c** Representative confocal images of PLA between hnRNPA2B1 and ALIX (red signal) in circTLCD4-RWDD3-overexpressing A549 cells with or without mutating K108 residue of hnRNPA2B1. Scale bars, 5 μm. **d** Schematic illustrating conserved SIM in ALIX sequence. **e** Western blotting analysis after co-IP assays to investigate the interaction between hnRNPA2B1 and ALIX after mutating K108 residue of hnRNPA2B1 or SIM in ALIX. **f** Confocal images of PLA between hnRNPA2B1 and ALIX (red signal) in A549 cells with hnRNPA2B1^K108R^ or mutation of the SIM in ALIX. Scale bars, 5 μm. **g** Western blotting analysis after co-IP assays to assess the activation of ALIX in A549 cells with hnRNPA2B1^K108R^ or mutation of the SIM in ALIX. **h** Representative confocal images of the co-localization between hnRNPA2B1 and the indicated subcellular markers in circTLCD4-RWDD3-overexpressing A549 cells with or without SENP3 treatment. Scale bars, 5 μm. **i** Pearson correlation coefficients were calculated from the A549 cells expressing hnRNPA2B1 and the indicated subcellular markers. **j** qRT-PCR analysis for circTLCD4-RWDD3 expression in EVs derived from circTLCD4-RWDD3-overexpressing A549 cells with or without knocking down ALIX. **k**, **l** Western blotting analysis for hnRNPA2B1 expression in circTLCD4-RWDD3-overexpressing A549 cells with or without knocking down ALIX (**k**) or in EVs derived from the indicated A549 cells (**l**). **m** qRT-PCR analysis for circTLCD4-RWDD3 expression in EVs derived from A549 cells with hnRNPA2B1^K108R^ or mutation of the SIM in ALIX. **n** Western blotting analysis of hnRNPA2B1 expression in EVs derived from A549 cells with hnRNPA2B1^K108R^ or mutation of the SIM in ALIX. **o** Detection of intracellular co-localization of ALIX and CHMP4B in A549 cells with hnRNPA2B1^K108R^ or mutation of the SIM in ALIX. Scale bars, 5 μm. **p** Pearson correlation coefficients were calculated from the indicated A549 cells expressing ALIX and CHMP4B. **q** qRT-PCR analysis for circTLCD4-RWDD3 expression in EVs derived from A549 cells with mutation of the SIM in ALIX or knocking down CHMP4B. **r** Western blotting analysis of hnRNPA2B1 expression in EVs derived from A549 cells with mutation of the SIM in ALIX or knocking down CHMP4B. The statistical difference was assessed by the unpaired Student’s *t*-test in (**j**); and one-way ANOVA followed by Dunnett tests in (**i**, **m**, **p**, and **q**). Error bars show the SD from three independent experiments. *P < 0.05; **P < 0.01

It has been widely reported that the attachment with SUMOs extends the ability of proteins to interplay with other proteins, thus exhibiting diverse biological functions in cancer progression.^25^ Therefore, we further detected the targeting proteins of circTLCD4-RWDD3-induced hnRNPA2B1 SUMOylation. Sliver staining after co-IP assays with anti-hnRNPA2B1 revealed an obvious band with a molecular weight on 90–100 kDa in hnRNPA2B1^WT^ group compared with hnRNPA2B1^K108^ group (Fig. 5a), which was then subjected into MS analysis. Among the potential proteins identified by MS analysis (Supplementary Table 5), ALIX was confirmed as the most interacted protein with SUMOylated hnRNPA2B1 by western blotting analysis, in which circTLCD4-RWDD3 overexpression significantly promoted the interaction between hnRNPA2B1 and ALIX, while mutating the K108 residue of hnRNPA2B1 abolished this effect (Fig. 5b, Supplementary Fig. 4w). Additionally, high levels of proximity ligation assay (PLA) signals showing the interaction between hnRNPA2B1 and ALIX were observed in the cytoplasm of circTLCD4-RWDD3 overexpressing NSCLC cells compared to the control, which were reduced by hnRNPA2B1 K108 residue mutation (Fig. 5c), indicating that circTLCD4-RWDD3-induced SUMOylation of hnRNPA2B1 is required for its interaction with ALIX.

To further investigate the molecular mechanism underlying the interaction between SUMOylated hnRNPA2B1 and ALIX, we performed sequence analysis for ALIX and identified a “V/I-X-V/I-V/I” SIM conserved in orthologs from zebrafish to mammals^30^ (Fig. 5d). Previous studies have reported that SUMOylation enables protein to establish noncovalent interaction with other proteins containing SIM.^17,31^ Thus, co-IP assays were conducted to validate whether ALIX directly interacts with hnRNPA2B1 through recognizing the SUMO2 modification on hnRNPA2B1 via SIM. The results showed that both hnRNPA2B1^K108R^ and the mutation of the SIM in ALIX (substitution of valine or isoleucine with alanine) contributed to the abrogation of the interaction between hnRNPA2B1 and ALIX (Fig. 5e). These results were confirmed in PLA (Fig. 5f), suggesting that the interaction between hnRNPA2B1 and ALIX relies on the hnRNPA2B1 SUMOylation and SIM in ALIX.

Considering that SIMs are required to recruit and activate the downstream effectors of SUMOylated proteins,^25,32^ we further explored whether circTLCD4-RWDD3-mediated SUMOylation of hnRNPA2B1 regulated the functional activation of ALIX by interacting with its SIM. The co-IP assays were conducted using 2H12 anti-ALIX antibodies, which specifically recognized the functional activated form of ALIX.^33,34^ The results showed that mutations the SUMO2 modification site of hnRNPA2B1, SIM alterations in ALIX, or overexpression of SENP3, hampered the immunoprecipitation of ALIX by 2H12 anti-ALIX antibodies (Fig. 5g, Supplementary Fig. 4x), demonstrating that the interaction between SUMOylated hnRNPA2B1 and ALIX via SIM significantly activates ALIX. Collectively, these results demonstrate that circTLCD4-RWDD3-induced SUMOylation on hnRNPA2B1 endows its ability to interact with ALIX and stimulate the functional activation of ALIX.

### SUMOylated hnRNPA2B1-activated ALIX triggers circTLCD4-RWDD3 loading into EVs in an ESCRT-III-dependent manner

ALIX served as the ESCRT accessory protein which plays a pivotal role in intraluminal vesicles (ILVs) formation within late endosomes and in sorting cargos into EVs during the endosomal trafficking process.^35^ The observation that circTLCD4-RWDD3 significantly induced the SUMOylation of hnRNPA2B1 to directly interact and drive the functional activation of ALIX prompted us to hypothesize that activated ALIX might mediate the sorting of circTLCD4-RWDD3 and hnRNPA2B1 into EVs during formation of late endosome. To confirm this hypothesis, immunofluorescence assays were conducted and revealed that a high co-localization between hnRNPA2B1 and Rab7 or CD63-indicated late endosome whereas only a minor proportion of hnRNPA2B1 co-localized with TGN46-indicated *trans*-Golgi and Rab5-indicated early endosome (Fig. 5h). Moreover, the Pearson correlation coefficient between hnRNPA2B1 and late endosome markers significantly increased upon upregulation of circTLCD4-RWDD3 expression in NSCLC cells, while overexpression of SENP3 markedly reduced this elevation induced by circTLCD4-RWDD3 overexpression (Fig. 5i), indicating that the sorting of hnRNPA2B1 into EVs might occur during ALIX-involved formation of late endosome and is regulated by circTLCD4-RWDD3-mediated SUMOylation. Additionally, the EVs from the CM of NSCLC cells with ALIX knocking down were isolated and subjected into evaluation circTLCD4-RWDD3 and hnRNPA2B1 expression. The results showed that knocking down ALIX greatly decreased the expression levels of circTLCD4-RWDD3 and hnRNPA2B1 in NSCLC cell-derived EVs without affecting the expression of hnRNPA2B1 in NSCLC cells (Fig. 5j–l), confirming that ALIX is involved in mediating the loading of circTLCD4-RWDD3 and hnRNPA2B1 into NSCLC cell-secreted EVs. Moreover, mutating the K108 residue of hnRNPA2B1 or SIM in ALIX significantly impaired the enrichment of circTLCD4-RWDD3 and hnRNPA2B1 in NSCLC cell-secreted EVs (Fig. 5m, n), further confirming that SUMOylated hnRNPA2B1 activates ALIX to package circTLCD4-RWDD3 and hnRNPA2B1 into NSCLC cell-secreted EVs.

Previous study has indicated that ALIX sorts cargos into EVs by recruiting ESCRT-III.^34^ Therefore, we further performed the immunofluorescence assays to investigate whether SUMOylated hnRNPA2B1 activated ALIX to recruit ESCRT-III and stimulate the sorting of circTLCD4-RWDD3 and hnRNPA2B1 into EVs. The results revealed that inhibiting the interaction between SUMOylated hnRNPA2B1 and SIM in ALIX impeded the co-localization of ALIX with charged multivesicular body protein 4B (CHMP4B), a component of ESCRT-III (Fig. 5o, p). Moreover, mutating ALIX SIM or downregulating CHMP4B greatly impaired the expression levels of circTLCD4-RWDD3 and SUMOylated hnRNPA2B1 in EVs (Fig. 5q, r, Supplementary Fig. 4y), indicating that SUMOylated hnRNPA2B1 activates ALIX to recruit ESCRT-III and facilitate the sorting of circTLCD4-RWDD3 and SUMOylated hnRNPA2B1 into NSCLC cell-derived EVs. Therefore, our data elucidate that the SUMOylated hnRNPA2B1 activates ALIX to recruit ESCRT-III, thereby drive the packaging of circTLCD4-RWDD3 and hnRNPA2B1 into EVs secreted by NSCLC cells.

### EV-packaged circTLCD4-RWDD3 is delivered to HLECs to facilitate lymphangiogenesis

**Fig. 6:**
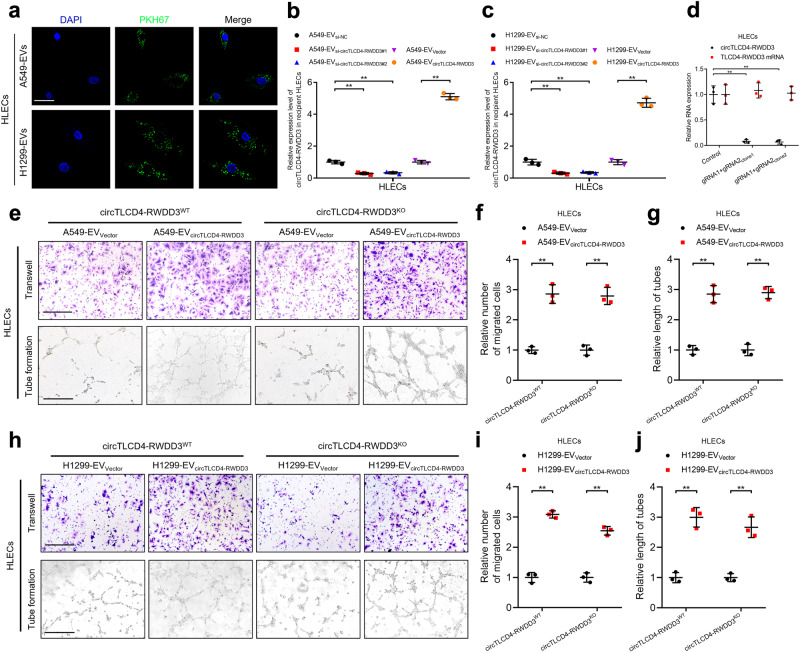
EV-packaged circTLCD4-RWDD3 is internalized by HLECs to facilitate lymphangiogenesis. **a** Representative fluorescence images of HLECs after incubating with PKH67-labeled EVs. Scale bars, 5 μm. **b**, **c** qRT-PCR analysis of circTLCD4-RWDD3 expression in HLECs treated with EVs. **d** qRT-PCR analysis of circTLCD4-RWDD3 and *TLCD4-RWDD3* mRNA expression in circTLCD4-RWDD3^KO^ HLECs. **e–g** Representative images (**e**) and quantification of the Transwell migration (**f**) and tube formation (**g**) of circTLCD4-RWDD3^WT^ and circTLCD4-RWDD3^KO^ HLECs treated with A549-EV_Vector_ or A549-EV_circTLCD4-RWDD3_. Scale bars, 100 µm. **h–j** Representative images (**h**) and quantification of the Transwell migration (**i**) and tube formation (**j**) of circTLCD4-RWDD3^WT^ and circTLCD4-RWDD3^KO^ HLECs treated with H1299-EV_Vector_ or H1299-EV_circTLCD4-RWDD3_. Scale bars, 100 µm. The statistical difference was assessed with one-way ANOVA followed by Dunnett tests in **b–d**; and unpaired Student’s *t*-test in (**b**, **c**, **f**, **g**, **i**, and **j**). Error bars show the SD from three independent experiments. *P < 0.05; **P < 0.01

As our results indicated that EV-packaged circTLCD4-RWDD3 enhanced lymphangiogenesis in NSCLC, we further explored the underlying mechanism. HLECs were incubated with NSCLC cell-derived EVs labeled with PKH67, thereby green punctate fluorescence signals from the PKH67-labeled EVs were observed in the cytoplasm of HLECs using confocal microscopy (Fig. 6a), indicating the internalization of NSCLC cell-derived EVs by HLECs. Additionally, treating with EVs from NSCLC cells increased the expression of circTLCD4-RWDD3 in HLECs compared to those treated with PBS (Supplementary Fig. 5a, b). Moreover, the expression of circTLCD4-RWDD3 in HLECs was greatly decreased through incubation with A549- or H1299-EV_si-circTLCD4-RWDD3_ compared with the control, while upregulated circTLCD4-RWDD3 expression was detected in HLECs treated with EVs secreted by A549 and H1299 cells with circTLCD4-RWDD3 overexpression (Fig. 6b, c), demonstrating that EV-packaged circTLCD4-RWDD3 is internalized by HLECs.

To exclude the possibility that the lymphangiogenesis was facilitated by activation of endogenous circTLCD4-RWDD3 in HLECs, we suppressed the formation of endogenous circTLCD4-RWDD3 in HLECs using CRISPR/Cas9 approach (Fig. 6d). Next, we investigated the effects of EV-packaged circTLCD4-RWDD3 on both circTLCD4-RWDD3-wild-type (circTLCD4-RWDD3^WT^) HLECs and circTLCD4-RWDD3^KO^ HLECs. Consistent with circTLCD4-RWDD3^WT^ HLECs, the tube formation and migratory ability of circTLCD4-RWDD3^KO^ HLECs was enhanced by EV-packaged circTLCD4-RWDD3 overexpression, whereas EVs derived from circTLCD4-RWDD3 knockdown NSCLC cells suppressed tube formation and migration of circTLCD4-RWDD3^KO^ HLECs (Fig. 6e–j, Supplementary Fig. 5c, d), suggesting that NSCLC cell-secreted EVs facilitate the lymphangiogenesis by transporting EV-packaged circTLCD4-RWDD3 rather than by activating endogenous circTLCD4-RWDD3 circularization in HLECs. These results suggest that NSCLC cells promote lymphangiogenesis through transmitting of EV-packaged circTLCD4-RWDD3 to HLECs.

### EV-packaged circTLCD4-RWDD3 upregulates PROX1 expression in HLECs

**Fig. 7:**
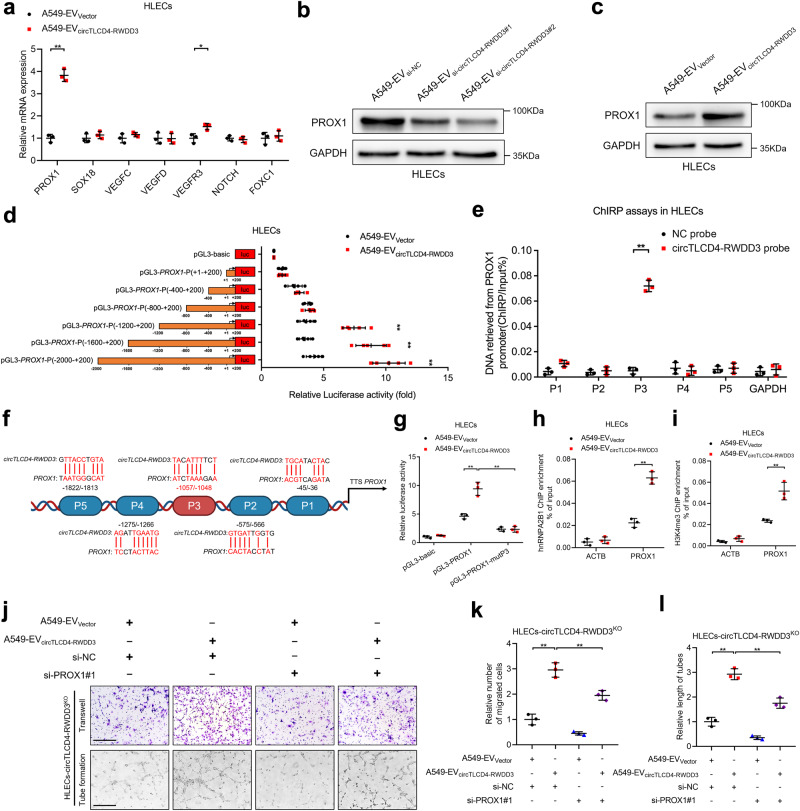
EV-packaged circTLCD4-RWDD3 promotes PROX1 expression in HLECs. **a** qRT-PCR analysis of lymphangiogenesis-related gene expression in A549-EV_Vector_- or A549-EV_circTLCD4-RWDD3_-treated HLECs. **b** Western blotting analysis to verify PROX1 expression in HLECs treated with A549-EV_si-NC_, A549-EV_si-circTLCD4-RWDD3#1_, or A549-EV_si-circTLCD4-RWDD3#2_. **c** Western blotting analysis to verify PROX1 expression in HLECs treated with A549-EV_Vector_ or A549-EV_circTLCD4-RWDD3_. **d** Transcriptional activity of *PROX1* in A549-EV_circTLCD4-RWDD3_-treated HLECs transfected with truncated *PROX1* promoter luciferase plasmids. **e** ChIRP assays to investigate the circTLCD4-RWDD3-associated chromatin fragments of the *PROX1* promoter in HLECs. **f** Schematic representation of the DNA-RNA triplex structure between circTLCD4-RWDD3 and the *PROX1* promoter. Schematic was created with BioRender (www.biorender.com). **g** Luciferase activity detected in A549-EV_Vector_- or A549-EV_circTLCD4-RWDD3_-treated HLECs with or without mutating the circTLCD4-RWDD3-binding site on *PROX1* promoter. **h**, **i** ChIP-qPCR of the enrichment of hnRNPA2B1 (**h**) and H3K4me3 (**i**) on *PROX1* promoter in A549-EV_Vector_- or A549-EV_circTLCD4-RWDD3_-treated HLECs. **j–l** Representative images (**j**) and quantification of Transwell migration (**k**) and tube formation (**l**) of A549-EV_Vector_- or A549-EV_circTLCD4-RWDD3_-treated circTLCD4-RWDD3^KO^ HLECs with or without knocking down PROX1. Scale bars, 100 µm. The statistical difference was assessed by the unpaired Student’s *t*-test in (**a**, **d**, **e**, **h**, and **i**); and one-way ANOVA followed by Dunnett tests in (**g**, **k**, and **l**). Error bars show the SD from three independent experiments. *P < 0.05; **P < 0.01

To explore the potential mechanism of EV-packaged circTLCD4-RWDD3 in facilitating HLECs tube formation and migration, we investigated the expression of lymphangiogenesis-related genes in HLECs incubated with EV-packaged circTLCD4-RWDD3. Subsequently, PROX1 was identified as the most significantly upregulated gene in HLECs treated with EVs secreted by NSCLC cells with circTLCD4-RWDD3 overexpression compared with the control group (Fig. 7a, Supplementary Fig. 5e–g), while PROX1 was greatly downregulated in HLECs treated with A549- or H1299-EV_si-circTLCD4-RWDD3_ compared with the control group (Supplementary Fig. 5h, i). Moreover, western blotting analysis showed that PROX1 expression was elevated in A549- or H1299-EV_circTLCD4-RWDD3_ group compared with the control, while HLECs treated with EVs derived from circTLCD4-RWDD3-downregulating NSCLC cells exhibited attenuated PROX1 expression compared with the control group (Fig. 7b, c, Supplementary Fig. 5j, k). It is well-recognized that PROX1 is considered as the core regulator for the specification of HLECs fate and the maintenance of HLECs identity.^6,36^ To elucidate the specific mechanism by which circTLCD4-RWDD3 regulated PROX1 expression in HLECs, we generated a series of luciferase constructs containing truncated *PROX1* promoter sequences locating from nucleotides −2000 upstream to +200 downstream of the transcriptional start site. These constructs were employed to assess whether EV-packaged circTLCD4-RWDD3 played the regulatory role in transcriptional activity of PROX1 in HLECs. Luciferase assays indicated that EV-packaged circTLCD4-RWDD3 enhanced transcriptional activity of PROX1 in HLECs by interacting with nucleotides −1200 to −800 region of *PROX1* promoter, which we narrowed down to a region from nucleotides −1057 to −1048 (referred to as P3) based on ChIRP assays and sequence alignment (Fig. 7d–f, Supplementary Fig. 5l). Mutation of this region prevented EV-packaged circTLCD4-RWDD3 to stimulate luciferase activity on *PROX1* promoter (Fig. 7g, Supplementary Fig. 5m). Moreover, ChIP assays showed that EVs from NSCLC cells overexpressing circTLCD4-RWDD3 led to significantly higher levels of hnRNPA2B1 and H3K4me3 at the *PROX1* promoter in HLECs than EVs from NSCLC cells expressing normal levels of circTLCD4-RWDD3 (Fig. 7h, i, Supplementary Fig. 5n–s), indicating that EV-packaged circTLCD4-RWDD3 increased hnRNPA2B1-induced H3K4me3 levels at the *PROX1* promoter in HLECs. The essential role of PROX1 in the lymphangiogenesis stimulated by EV-packaged circTLCD4-RWDD3 was confirmed by showing that knocking down of PROX1 strongly weakened the lymphangiogenesis induced by EVs from NSCLC cells overexpressing the circTLCD4-RWDD3 (Fig. 7j–l, Supplementary Fig. 6a–d). Together, these results suggest that EV-packaged circTLCD4-RWDD3 enhances transcriptional activity of PROX1 expression to facilitate NSCLC lymphangiogenesis.

### Inhibiting delivery of circTLCD4-RWDD3 by EVs reduces LN metastasis of NSCLC

Considering that SUMOylated hnRNPA2B1 was crucial in packaging circTLCD4-RWDD3 into NSCLC cells-derived EVs, we investigated the effect of impairing hnRNPA2B1 SUMOylation on tube formation and migration of HLECs facilitated by EV-packaged circTLCD4-RWDD3. The results showed that mutating K108 residue of hnRNPA2B1 to inhibit hnRNPA2B1 SUMOylation significantly suppressed the ability of circTLCD4-RWDD3-overexpressing NSCLC cells-derived EVs to induce the tube formation and migration of HLECs compared with the control (Supplementary Fig. 6e). Moreover, we further investigated the role of hnRNPA2B1 SUMOylation on LN metastasis of NSCLC induced by EV-packaged circTLCD4-RWDD3 in vivo. *IVIS* imaging revealed that mutating K108 residue of hnRNPA2B1 markedly suppressed the metastasis of NSCLC cells to the popliteal LNs of the nude mice compared with A549-EV_circTLCD4-RWDD3+hnRNPA2B1_^WT^ group (Supplementary Fig. 6f, g). As shown in Supplementary Fig. 6h, i, the volume of popliteal LNs and popliteal LN metastasis rate were significantly increased in mice injected with circTLCD4-RWDD3-overexpressing NSCLC cell-derived EVs compared with the control group, whereas inhibiting the SUMO2 modification on hnRNPA2B1 by mutating K108 residue of hnRNPA2B1 attenuated this effect, indicating that SUMOylated hnRNPA2B1-mediated package of circTLCD4-RWDD3 into NSCLC cells-derived EVs is essential for the LN metastasis of NSCLC in vivo.

**Fig. 8:**
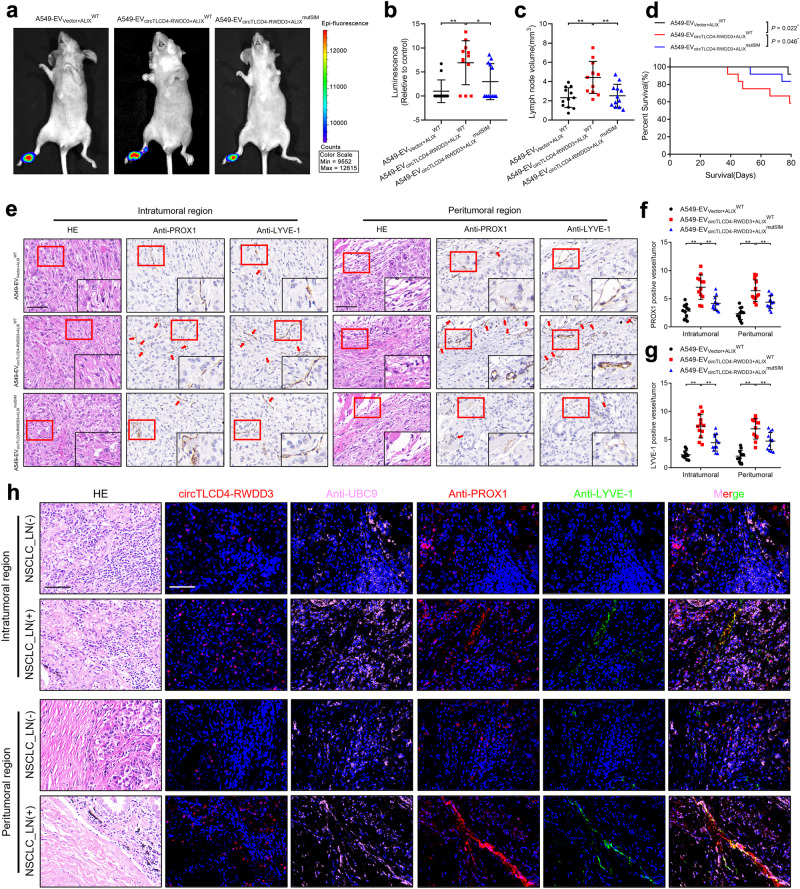
Blocking the transmission of EV-packaged circTLCD4-RWDD3 suppresses LN metastasis of NSCLC. **a**, **b** Representative bioluminescence images (**a**) and quantification (**b**) of popliteal metastatic LNs from nude mice treated with EVs secreted by control or circTLCD4-RWDD3-overexpressing A549 cells with or without mutating SIM in ALIX (*n* = 12 per group). (**c**) Quantification of popliteal LN volume of nude mice treated with EVs (*n* = 12 per group). **d** Kaplan–Meier curves show the survival of nude mice treated with EVs secreted by control or circTLCD4-RWDD3-overexpressing A549 cells with or without mutating SIM in ALIX. **e–g** Representative IHC images and percentages of PROX1- or LYVE-1-indicated lymphatic vessel density in footpad primary tumor tissues. Scale bars, 50 µm. **h** Representative fluorescence images to confirm the correlation for circTLCD4-RWDD3 expression, UBC9 and PROX1 expression, and LYVE-1-indicated MLD in the serial section of NSCLC tissues from the multicenter cohorts (*n* = 312). Scale bars, 50 μm. The statistical difference was assessed through one-way ANOVA followed by Dunnett tests in (**b**, **c**, **f**, and **g**). Error bars show the SD from three independent experiments. *P < 0.05; **P < 0.01

Since the SIM in ALIX plays a pivotal role in binding with SUMOylated hnRNPA2B1 to further mediate packaging circTLCD4-RWDD3 into EVs and trigger the LN metastasis of NSCLC, we further sought to examine whether mutating ALIX SIM could suppress NSCLC lymphangiogenesis and LN metastasis facilitated by EV-packaged circTLCD4-RWDD3. Transwell migration and tube formation assays revealed that EVs derived from circTLCD4-RWDD3-overexpressing NSCLC cells notably enhanced lymphangiogenesis in vitro, whereas mutation of SIM in ALIX reversed the lymphangiogenesis induced by EV-packaged circTLCD4-RWDD3 (Supplementary Fig. 6j), suggesting that ALIX SIM acts crucial role in EV-packaged circTLCD4-RWDD3-induced lymphangiogenesis in vitro. Additionally, we constructed popliteal LN metastasis model and intratumorally inoculated EVs secreted by NSCLC cells in diverse groups. *IVIS* imaging showed that mutating ALIX SIM significantly inhibited popliteal LN metastasis promoted by injection of A549-EV_circTLCD4-RWDD3+ALIX_^WT^ (Fig. 8a, b). Lower volume of popliteal LNs was obtained from the A549-EV_circTLCD4-RWDD3+ALIX_^mutSIM^ group than A549-EV_circTLCD4-RWDD3+ALIX_^WT^ group (Fig. 8c). Moreover, mice in the A549-EV_circTLCD4-RWDD3+ALIX_^mutSIM^ group had prolonged survival time compared with those in the A549-EV_circTLCD4-RWDD3+ALIX_^WT^ group (Fig. 8d). Immunohistochemical staining with anti-PROX1 or anti-LYVE-1 antibodies consistently revealed increased number of lymphatic vessels in footpad tumors of mice treated with A549-EV_circTLCD4-RWDD3+ALIX_^WT^ compared with control group, while mutating ALIX SIM contributed to significant reduction in the number of EV-packaged circTLCD4-RWDD3-induced lymphatic vessels (Fig. 8e–g), indicating that lymphangiogenesis induced by EV-packaged circTLCD4-RWDD3 could be impaired by the mutation of SIM in ALIX in NSCLC cells. Collectively, these results demonstrate that ALIX SIM is indispensable for NSCLC lymphangiogenesis and LN metastasis facilitated by EV-packaged circTLCD4-RWDD3.

### Clinical relevance of circTLCD4-RWDD3/UBC9/PROX1 axis in the LN metastasis of NSCLC

**Fig. 9:**
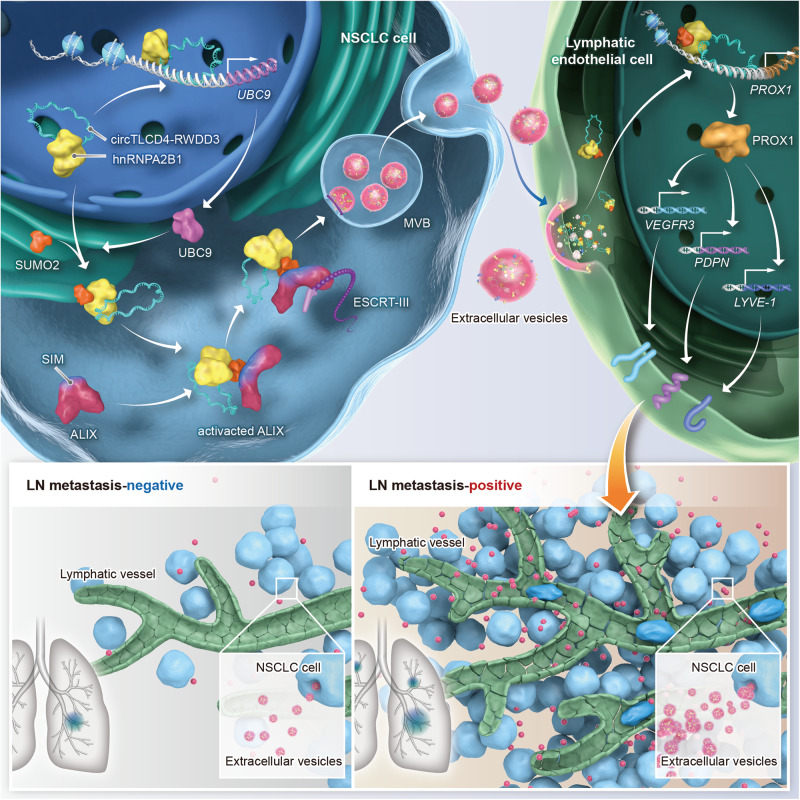
Schematic illustrating the potential mechanism by which EV-packaged circTLCD4-RWDD3 promotes LN metastasis of NSCLC

Given that EV-packaged lncRNAs were widely proved as promising diagnostic biomarkers in various cancers, we examined the expression level of circTLCD4-RWDD3 in serum EVs derived from LN metastasis-positive and LN metastasis-negative NSCLC patients. The results showed that the expression of circTLCD4-RWDD3 in serum EVs from NSCLC patients with LN metastasis was higher than those from patients without LN metastasis (Supplementary Fig. 6k–m, Supplementary Table 6). We have determined that circTLCD4-RWDD3 promoted UBC9 expression to facilitate SUMOylation of hnRNPA2B1 and mediate the sorting of circTLCD4-RWDD3 into EVs, which were transported to HLECs to induce lymphangiogenesis via PROX1 overexpression. Thus, we further assess the clinical relevance of circTLCD4-RWDD3/UBC9/PROX1 axis-mediated lymphangiogenesis and LN metastasis in the multicenter NSCLC cohort. As shown in Fig. 8h and Supplementary Fig. 6n, overexpression of circTLCD4-RWDD3 correlated with upregulated UBC9 expression in NSCLC tissues. Moreover, increased number of LYVE-1- and PROX1-labeled lymphatic vessels were observed in both intratumoral and peritumoral region of NSCLC with LN metastasis. Importantly, NSCLC tissues with high expression of circTLCD4-RWDD3 and UBC9 exhibited elevated LYVE-1- and PROX1-labeled MLD. Together, these findings indicate that unregulated circTLCD4-RWDD3 increases UBC9 expression to induce the SUMOylation of hnRNPA2B1 and enhance its sorting into EVs, eventually enhancing lymphangiogenesis and LN metastasis of NSCLC (Fig. 9).

## Discussion

LN metastasis represents a critical determinant in determining prognosis and guiding treatment strategies for patients with NSCLC, largely due to its association with a high risk of disease recurrence.^37^ Thus, investigations of the molecular mechanisms underlying LN metastasis in NSCLC and identification of novel LN metastasis-associated targets are urgently warranted for NSCLC prevention and treatment. Herein, we provided evidence from cell culture experiments, a mouse model, and data from a large multicenter patient cohort, demonstrating that EV-packaged circRNA, termed circTLCD4-RWDD3, can be internalized by HLECs, where the circTLCD4-RWDD3 induced lymphangiogenesis and facilitated LN metastasis in NSCLC. We demonstrated that circTLCD4-RWDD3 bound with the nucleotides −259 to −250 of *UBC9* promoter to form a DNA-RNA triplex and further activated *UBC9* transcription by recruiting hnRNPA2B1 and H3K4me3 in NSCLC cells. Moreover, circTLCD4-RWDD3-mediated UBC9 overexpression promoted SUMO2 modification on K108 residue of hnRNPA2B1, which interacted noncovalently with the SIM on ALIX to load circTLCD4-RWDD3 into EVs derived from NSCLC cells. Subsequently, EV-packaged circTLCD4-RWDD3 was internalized by HLECs to facilitate PROX1 expression by interacting with the nucleotides −1057 to −1048 of *PROX1* promoter to induce hnRNPA2B1-mediated H3K4me3, thereby enhancing lymphangiogenesis in NSCLC. This study represents the comprehensive exploration of the mechanisms underlying lymphangiogenesis and LN metastasis in NSCLC modulated by EV-packaged circRNA, suggesting that targeting EV-packaged circTLCD4-RWDD3 is a promising strategy for NSCLC patients with LN metastasis.

Our work is consistent with several previous studies which have shown that hnRNPA2B1 plays a role in the packaging of non-coding RNA into EVs.^12,38^ It has been reported that the specific motifs, such as GGAG, served as a determinant for hnRNPA2B1 in regulating the localization of non-coding RNA into EVs.^13,39,40^ However, this well-established motif does not appear to be involved in the sorting of circTLCD4-RWDD3. SUMOylation is a dynamic and reversible biological event that possesses ability to amplify the effects and signals of targeting proteins through cascade reaction.^25^ Instead, it has been reported that hnRNPA2B1 was widely modified with SUMO modifiers, thus allowing it to engage in diverse biological functions during cancer progression.^17,40^ Consistently, we found that SUMOylation of hnRNPA2B1 was essential for the packaging of circTLCD4-RWDD3 into NSCLC-derived EVs in the present study. Unlike the previous study that hnRNPA2B1 was modified by SUMO1 at K108 site,^17^ we demonstrated that hnRNPA2B1 in NSCLC cells was modified at the K108 site with SUMO2, which mediated the interaction of hnRNPA2B1 with the SIM in ALIX and activated ALIX to recruit ESCRT-III, thus facilitating the packaging of circTLCD4-RWDD3 into EVs. Moreover, blocking the transportation of EV-packaged circTLCD4-RWDD3 by impairing hnRNPA2B1 SUMOylation or mutating ALIX SIM in NSCLC cells inhibited NSCLC lymphangiogenesis and LN metastasis in vitro and in vivo. Therefore, our findings demonstrate a novel mechanism that hnRNPA2B1-mediated loading of circTLCD4-RWDD3 into EVs by interacting with ALIX SIM in a SUMOylation-dependent manner, which may provide innovative strategies for the development of targeted drugs towards lymphatic metastasis in NSCLC.

ALIX is recognized for its role in membrane remodeling and cargo clustering during endosome maturation into multivesicular bodies.^35^ It is also acknowledged for its ability to become activated and recruit ESCRT to package various molecules into EVs.^34,41^ However, the precise mechanisms governing ALIX activation are unclear, and the present work defines at least one activation pathway: SUMOylated hnRNPA2B1 directly interacts with the “V/I-X-V/I-V/I” SIM in ALIX to induce the activation of ALIX. In addition, mutating the K108 SUMOylation modification site of hnRNPA2B1 or SIM in ALIX significantly inhibited the activation of ALIX and suppressed the biological capacity of ALIX to recruit ESCRT-III component, thereby impairing the enrichment of circTLCD4-RWDD3 in NSCLC cell-derived EVs. Our results highlight a SUMOylation-dependent pathway underlying the activation of ALIX to mediate the loading cargos into EVs, proposing a new perspective on ALIX-mediated the sorting of circRNAs into EVs.

The clinical data in present study are derived from a large multicenter cohort, which may make our findings more reliable. This is particularly important given the considerable heterogeneity in molecular characteristics and pathological manifestations observed in NSCLC,^42^ which pose a great challenge when seeking universal diagnostic or therapeutic targets for this disease. Herein, the pathological overexpression of EV-packaged circTLCD4-RWDD3 in LN metastatic NSCLC tissues were validated in multicenter cohort of 312-case NSCLC. Furthermore, we conduct univariate and multivariate Cox regression analysis to further demonstrate that circTLCD4-RWDD3 expression was an independent prognostic factor for predicting the OS and DFS of NSCLC patients. Importantly, our proposed axis involving circTLCD4-RWDD3, UBC9, and PROX1, is likely to be clinically relevant to LN metastasis of NSCLC. Therefore, our data provide reliable and comprehensive evidence supporting the potential therapeutic targeting of EV-packaged circTLCD4-RWDD3 as an avenue for addressing LN metastasis in NSCLC.

In summary, our study has illuminated a novel mechanism by which SUMOylated hnRNPA2B1 promotes ALIX activation to facilitate the packaging of circTLCD4-RWDD3 into EVs by interacting with the SIM motif in ALIX, resulting in the lymphangiogenesis and LN metastasis in NSCLC. The systematic elucidation of the mechanism of SUMOylation-dependent EV-packaged circTLCD4-RWDD3 in promoting the LN metastasis in NSCLC, suggesting EV-packaged circTLCD4-RWDD3 as an effective therapeutic target for LN metastatic NSCLC.

## Materials and methods

### Patient samples

A total of 312 pairs of NSCLC tissues and NATs were collected from patients who underwent surgical resection in the department of thoracic surgery at Peking Union Medical College Hospital of Chinese Academy of Medical Sciences (Beijing, China; 112 cases), Sun Yat-sen Memorial Hospital of Sun Yat-sen University (Guangzhou, China; 110 cases) and Sun Yat-sen University Cancer Center of Sun Yat-sen University (Guangzhou, China; 90 cases). All paired samples of tumor tissues and NATs were independently confirmed by two experienced histopathologists. The samples were obtained with informed consent, and the study was approved by Ethics Committee of each participating center (ZS-3327 from Peking Union Medical College Hospital, SYSKY-2023-1040-01 from Sun Yat-sen Memorial Hospital, and B2022-011-01 from Sun Yat-sen University Cancer Center). The study was conducted in accordance with recognized ethical guidelines. Patient and disease characteristics are summarized in Supplementary Tables 1 and 6.

### Cell lines and cell culture

Normal human bronchial epithelial cell line BEAS-2B was purchased from the American Type Culture Collection (ATCC, Manassas, VA, USA), together with the following human NSCLC cell lines: A549, HCC827, NCI-H1650, NCI-H1299, NCI-H1703, NCI-H520, and NCI-H460. HLECs were obtained from ScienCell Research Laboratories (San Diego, California, USA).

BEAS-2B cells were cultured in Dulbecco’s modified Eagle medium (DMEM; Gibco, catalog no. C11995500BT). A549 cells were cultured in Ham’s F-12K culture medium (Gibco, catalog no. 21127022), while the other NSCLC cell lines were cultured in RPMI 1640 culture medium (Gibco, catalog no. C11875500BT). Medium for all these cell lines was supplemented with 10% fetal bovine serum (FBS; BI, catalog no. 04-001-1ACS). HLECs were cultured in endothelial cell medium (ECM) supplemented with 5% FBS (ScienCell Research Laboratories, catalog no. 1001). All cultures were maintained at 37 °C in an incubator containing humidified air and atmosphere of 5% CO_2_.

### Mouse popliteal lymphatic metastasis model

BALB/c nude mice 4–5 weeks old and free of specific pathogens (Beijing Vital River Laboratories Animal Technology, Beijing, China) received an injection of 20 μL PBS containing mCherry-labeled A549 cells (5 × 10^5^) in the right footpad, followed by intratumorally injecting 10 μg isolated EVs suspended in 50 μL PBS every 3 days. Once weekly, the popliteal LNs were checked for metastasis using *IVIS* (Xenogen Corporation, Alameda, California). After the footpad tumors reached a volume of 200 mm^3^, the popliteal LNs and footpad primary tumors were collected and embedded with paraffin for further analysis by IHC analysis. Animal studies were conducted with the authorization of the Experimental Animal Welfare and Ethics Committee of Peking Union Medical College Hospital (XHDW-2022-040) and the animals were handled in accordance with institutional guidelines.

### EV internalization analysis

Isolated EVs were labeled with PKH67 using the PKH67 Green Fluorescent Cell Linker Mini Kit (Sigma-Aldrich, Germany, catalog no. mini67), then HLECs were incubated with 10 μg/mL PKH67-labeled EVs for 6 h at 37 °C in 5% CO_2_. Cultures were washed three times by PBS, fixed with 4% paraformaldehyde, and stained with DAPI. Images were captured using an LSM710 confocal microscope (Carl Zeiss).

### RNA pull-down analysis

NSCLC cells (2 × 10^7^) were lysed with 200 μL lysis buffer in the Magnetic RNA-Protein Pull-Down Kit (Thermo Scientific, catalog no. 20164), immediately frozen in liquid nitrogen, then stored at −80 °C for at least 2 h to ensure complete lysis. Lysate was centrifuged for 30 min at 12,000 *g*, and the supernatant was incubated at 4 °C overnight with streptavidin-labeled magnetic beads (Invitrogen, catalog no. 88817) that had previously been incubated for 30 min at room temperature with biotinylated probe against circTLCD4-RWDD3 (Genepharma) (50 μL beads per 50 pmol probe). After overnight incubation, the beads were washed five times with wash buffer, the proteins were eluted to be further analyzed by MS and western blotting analysis.

### Proximity ligation assay (PLA)

To examine the interaction between ALIX and SUMOylated hnRNPA2B1, the PLA was conducted. NSCLC (2 × 10^4^) cells were cultured on a confocal dish and fixed with 4% paraformaldehyde for 15 min. After blocking with 5% BSA at room temperature for 1 h, the PLA was conducted using Duolink PLA Multicolor Probemaker Kit-Far Red (Sigma-Aldrich, catalog no. DUO96040) with the primary antibodies against ALIX and hnRNPA2B1 according to the manufacturer’s instruction. The interactions between ALIX and SUMOylated hnRNPA2B1 was visualized and captured using LSM710 confocal microscope (Carl Zeiss).

### Statistical analysis

All statistical analyses were performed using GraphPad Prism 9 software (GraphPad Software, La Jolla, CA, USA) and SPSS 26.0 software (IBM, Chicago, IL, USA). Quantitative data were presented as mean ± SD of at least three independent experiments. The statistically differences were determined using either the unpaired Student’s *t*-test or the one-way ANOVA followed by Dunnett tests for continuous variables and with Chi-square test for categorical variables. Associations were explored using Pearson correlation analysis. OS and DFS between different groups were compared using the Kaplan-Meier analysis with the log-rank test. Univariate and multivariate Cox regression analysis were conducted to identify factors associated with survival. Differences associated with *P* < 0.05 were considered to be significant.

### Supplementary information

Supplementary Materials

## Data Availability

All data in the present study are available from the authors. The sequencing generated in this study are publicly available in the Gene Expression Omnibus (https://www.ncbi.nlm.nih.gov/geo/) under accession code GSE235634.
