# Supplementary material for: SUMOylation-triggered ALIX activation modulates extracellular vesicles circTLCD4-RWDD3 to promote lymphatic metastasis of non-small cell lung cancer
Source: Signal Transduct Target Ther. 2023 Nov 4;8:426. doi: 10.1038/s41392-023-01685-0 (PMC10625632; doi:10.1038/s41392-023-01685-0)
Supplement: Supplementary file 1 — Supplementary Materials [file 41392_2023_1685_MOESM1_ESM.docx]

**Supplementary Materials for**

**SUMOylation-triggered ALIX activation modulates extracellular vesicles circTLCD4-RWDD3 to promote lymphatic metastasis of non-small cell lung cancer**

Xiayao Diao ^1#^, Chao Guo ^1#^, Hanhao Zheng ^2,3#^, Ke Zhao ^1^, Yuming Luo ^4^, Mingjie An ^2,3^, Yan Lin ^2,3^, Jiancheng Chen ^2,3^, Yuanlong Li ^2,3^, Yuting Li ^5^, Xuehan Gao ^1^, Jiaqi Zhang ^1^, Mengxin Zhou ^1^, Wenliang Bai ^1^, Lei Liu ^1^, Guige Wang ^1^, Lanjun Zhang ^6^, Xiaotian He ^6^, Rusi Zhang ^6^, Zhihua Li ^7^, Changhao Chen ^2,3*^, Shanqing Li ^1*^

Correspondence to: chenchh53@mail.sysu.edu.cn (Changhao Chen), lishanqing@pumch.cn (Shanqing Li)

**This PDF file includes:**

Figures. S1 to S7

Tables S1 to S8

Materials and Methods

**Supplementary Figures and Figure Legends**

**
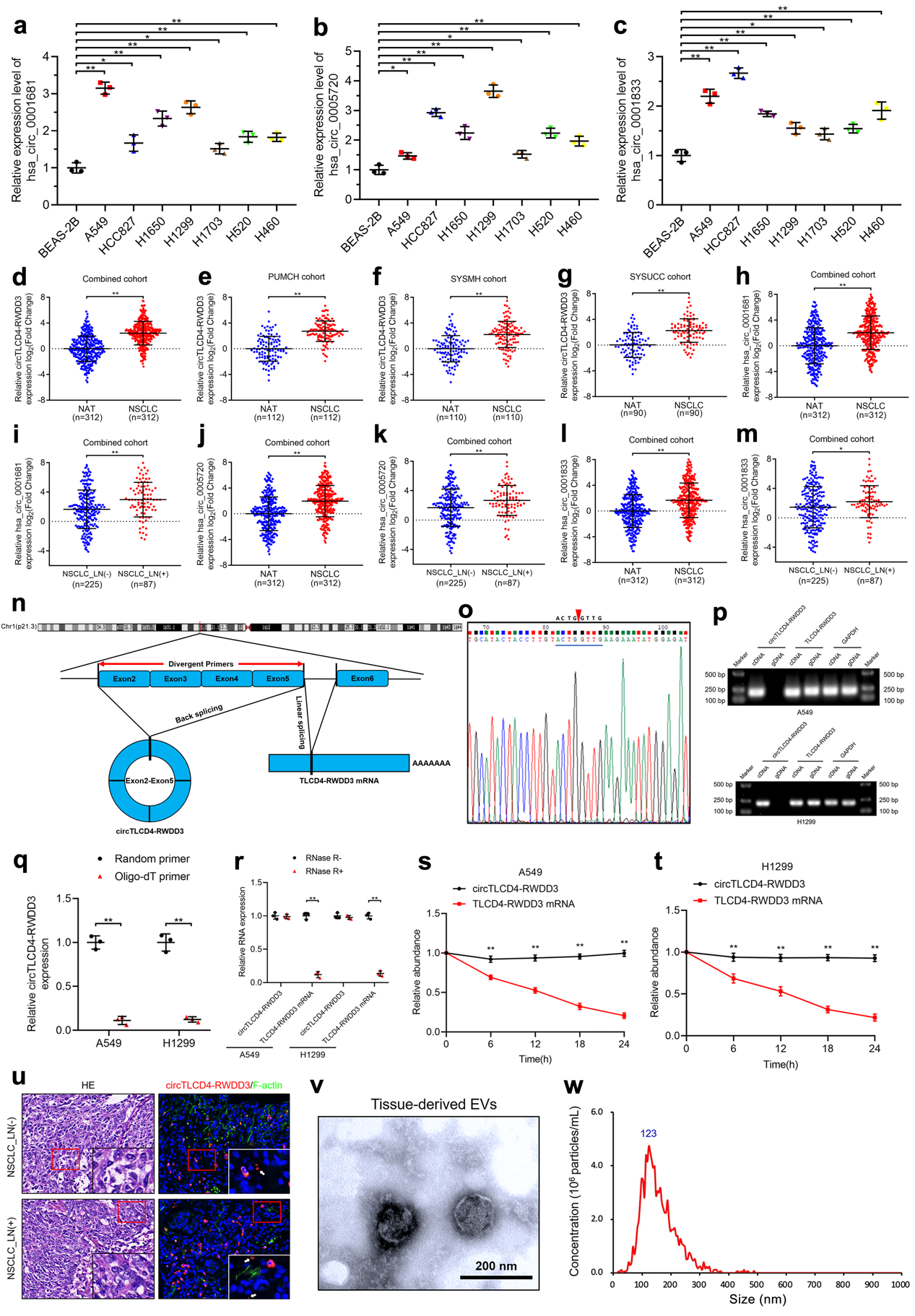
**

**Supplementary Figure S1. The identification and characterization of** **circTLCD4-RWDD3 in** **NSCLC.**

**(a-c)** qRT-PCR analysis of has_circ_0001681 **(a),** has_circ_0005720 **(b)** and has_circ_0001833 **(c)** expression in NSCLC cell lines and human bronchial epithelial cell line. **(d-g)** qRT-PCR analysis of circTLCD4-RWDD3 expression in NSCLC tissues and NATs from PUMCH, SYSMH, SYSUCC and combined cohort, respectively. **(h-i)** qRT-PCR analysis of has_circ_0001681 expression in NSCLC tissues and NATs **(h)** or in LN-positive and LN-negative NSCLC tissues **(i)** from combined multicenter cohort. **(j-k)** qRT-PCR analysis of has_circ_0005720 expression in NSCLC tissues and NATs **(j)** or in LN-positive and LN-negative NSCLC tissues **(k)** from combined multicenter cohort. **(l-m)** qRT-PCR analysis of has_circ_0001833 expression in NSCLC tissues and NATs **(l)** or in LN-positive and LN-negative NSCLC tissues **(m)** from combined multicenter cohort. **(n)** Schematic illustration showing the genomic loci of the *TLCD4-RWDD3* gene and the circTLCD4-RWDD3 derived from exon 2 to 5 of *TLCD4-RWDD3*. **(o)** Sanger sequencing for the back-splice junction site of circTLCD4-RWDD3. **(p)** PCR analysis for circTLCD4-RWDD3 and *TLCD4-RWDD3* in the cDNA and gDNA of A549 and H1299 cells. GAPDH was used as normal control. **(q)** qRT-PCR analysis of circTLCD4-RWDD3 expression using random primers or oligo-dT primers. **(r)** circTLCD4-RWDD3 and *TLCD4-RWDD3* expression analyzed by qRT-PCR in A549 and H1299 cells treated with RNase R. **(s-t)** Actinomycin D assays to assess the stability of circTLCD4-RWDD3 and *TLCD4-RWDD3* mRNA in A549 **(s)** and H1299 cells **(t)** at the indicated time points. **(u)** Representative images for RNA fluorescence in situ hybridization to investigate the extracellular expression of circTLCD4-RWDD3 (indicated by white arrows) in LN-positive NSCLC tissues compared with LN-negative NSCLC tissues. Scale bars, 50 μm. **(v-w)** TEM **(v)** and NTA **(w)** identified the characteristics of tissue-derived EVs. Scale bars, 200 nm. The statistical difference was assessed through one-way ANOVA followed by Dunnett tests in **a-c**; and nonparametric Mann-Whitney *U* test in **d-m**; and unpaired Student’s *t*-test in **q-t**. Error bars show the SD from three independent experiments. ^*^, *P* < 0.05; ^**^, *P* < 0.01.


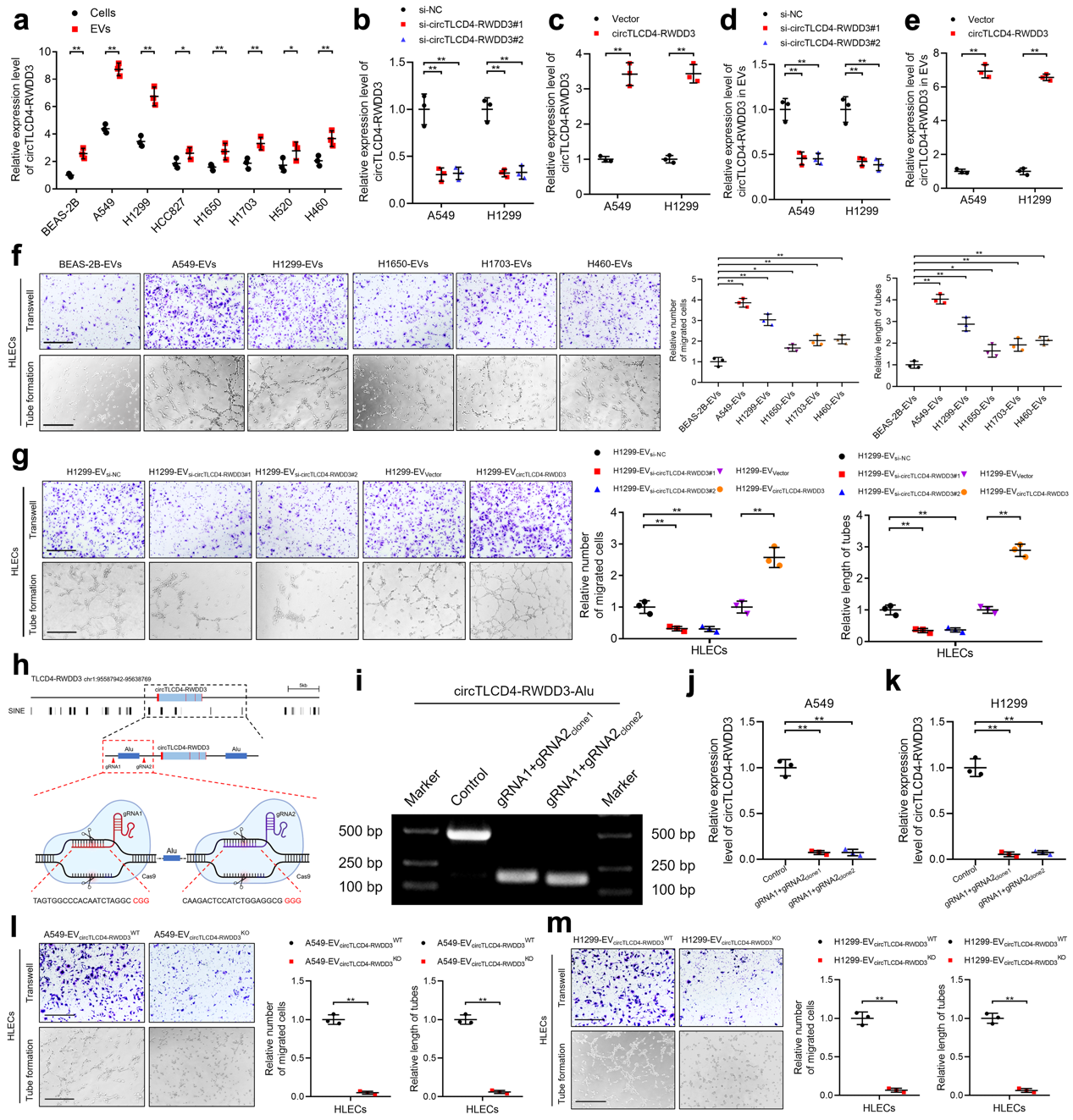


**Supplementary Figure S2. circTLCD4-RWDD3 is overexpressed in NSCLC cells-secreted EVs.**

**(a)** qRT-PCR analysis of circTLCD4-RWDD3 expression in indicated lung cancer cell lines and corresponding EVs. **(b-e)** qRT-PCR analysis of circTLCD4-RWDD3 expression in NSCLC cell lines and corresponding EVs after knocking down or overexpressing circTLCD4-RWDD3. **(f)** Representative images and quantification of tube formation and Transwell migration for HLECs treated with NSCLC cells or human bronchial epithelial cells-secreted EVs. Scale bars, 100μm. **(g)** Representative images and quantiﬁcation of the tube formation and Transwell migration of HLECs treated with circTLCD4-RWDD3-downregulated or - upregulated H1299 cell-derived EVs. Scale bars, 100 µm. **(h)** Schematic representation of CRISPR/Cas9-mediated circTLCD4-RWDD3 knockout. Schematic was created with BioRender (www.biorender.com). **(i)** PCR analysis to confirm CRISPR/Cas9-mediated the deletion of the proximal Alu element. **(j-k)** qRT-PCR analysis of circTLCD4-RWDD3 expression in circTLCD4-RWDD3^KO^ A549 **(j)** and H1299 cells **(k)**. **(l)** Representative images and quantification of tube formation and Transwell migration for HLECs treated with circTLCD4-RWDD3^KO^ A549 cells-secreted EVs. Scale bars, 100μm. **(m)** Representative images and quantification of tube formation and Transwell migration for HLECs treated with circTLCD4-RWDD3^KO^ H1299 cells-secreted EVs. Scale bars, 100μm. The statistical difference was assessed with one-way ANOVA followed by Dunnett tests in **b**, **d**, **f**, **g**, **j**, and **k**; and unpaired Student’s *t*-test in **a**, **c**, **e**, **g**, **l**, and **m**. Error bars show the SD from three independent experiments. ^*^, *P* < 0.05; ^**^, *P* < 0.01.


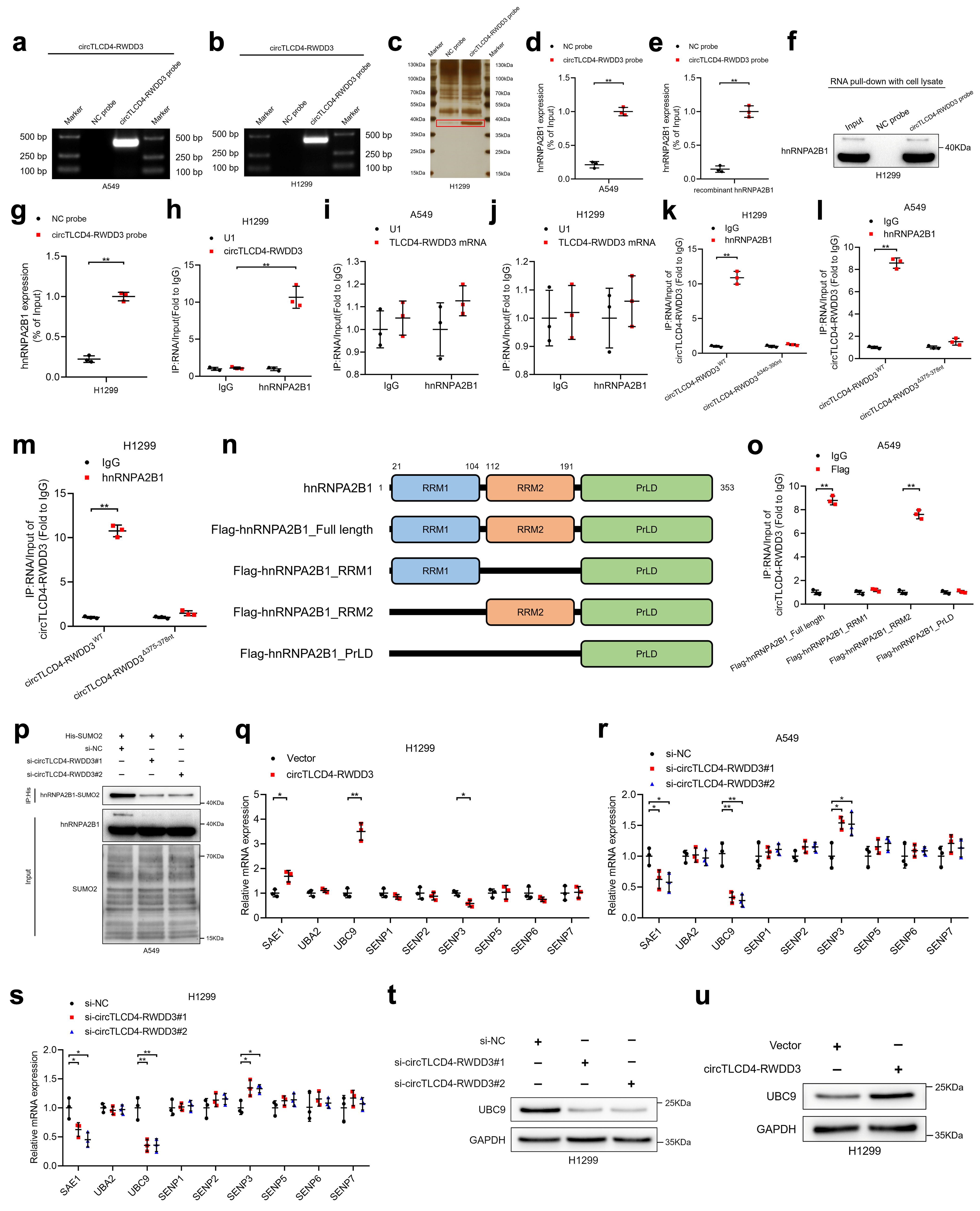


**Supplementary Figure S3.** **circTLCD4-RWDD3 promotes *UBC9* transcription by recruiting hnRNPA2B1.**

**(a-b)** PCR analysis to show circTLCD4-RWDD3 pull-down efficiency in RNA pull-down assays from A549 **(a)** and H1299-cell lysates **(b)** with circTLCD4-RWDD3 probe or NC probe. **(c)** Silver staining image of RNA pull-down assay with circTLCD4-RWDD3 and NC probes in H1299 cells. **(d-g)** Western blotting and quantification analysis of the interaction between circTLCD4-RWDD3 and hnRNPA2B1. **(h)** RIP assays revealed the enrichment of circTLCD4-RWDD3 by hnRNPA2B1 in H1299 cells. **(i-j)** RIP assays revealed that *TLCD4-RWDD3* mRNA did not enrich by hnRNPA2B1 in A549 **(i)** and H1299 cells **(j)**. **(k)** RIP assays after mutating the 340-390 nt regions of circTLCD4-RWDD3 in H1299 cells. **(l-m)** RIP assays after mutating the CAUU motif of circTLCD4-RWDD3 in A549 **(l)** and H1299 cells **(m)**. **(n)** Schematic representation of full length or domain-truncated fragments of hnRNPA2B1. RRM, RNA recognition motif; PrLD, prion-like domain. **(o)** RIP assays revealed the enrichment of circTLCD4-RWDD3 by Flag-tagged full length or indicated domain-truncated hnNRNPA2B1 in A549 cells. **(p)** Western blotting analysis of SUMO2 modification on hnRNPA2B1 in A549 cells with or without circTLCD4-RWDD3 downregulation. **(q)** qRT-PCR analysis of the SUMOylation-related enzymes in circTLCD4-RWDD3-overexpressing H1299 cells. **(r-s)** qRT-PCR analysis of the SUMOylation-related enzymes after circTLCD4-RWDD3-silencing in A549 **(r)** and H1299 cells **(s)**. **(t-u)** Western blotting analysis of UBC9 after upregulating **(t)** or downregulating circTLCD4-RWDD3 in H1299 cells **(u)**. The statistical difference was assessed with unpaired Student’s *t*-test in **d**, **e**, **g-m**, **o**, and **q**; and one-way ANOVA followed by Dunnett tests in **r** and **s**. Error bars show the SD from three independent experiments. ^*^, *P* < 0.05; ^**^, *P* < 0.01.


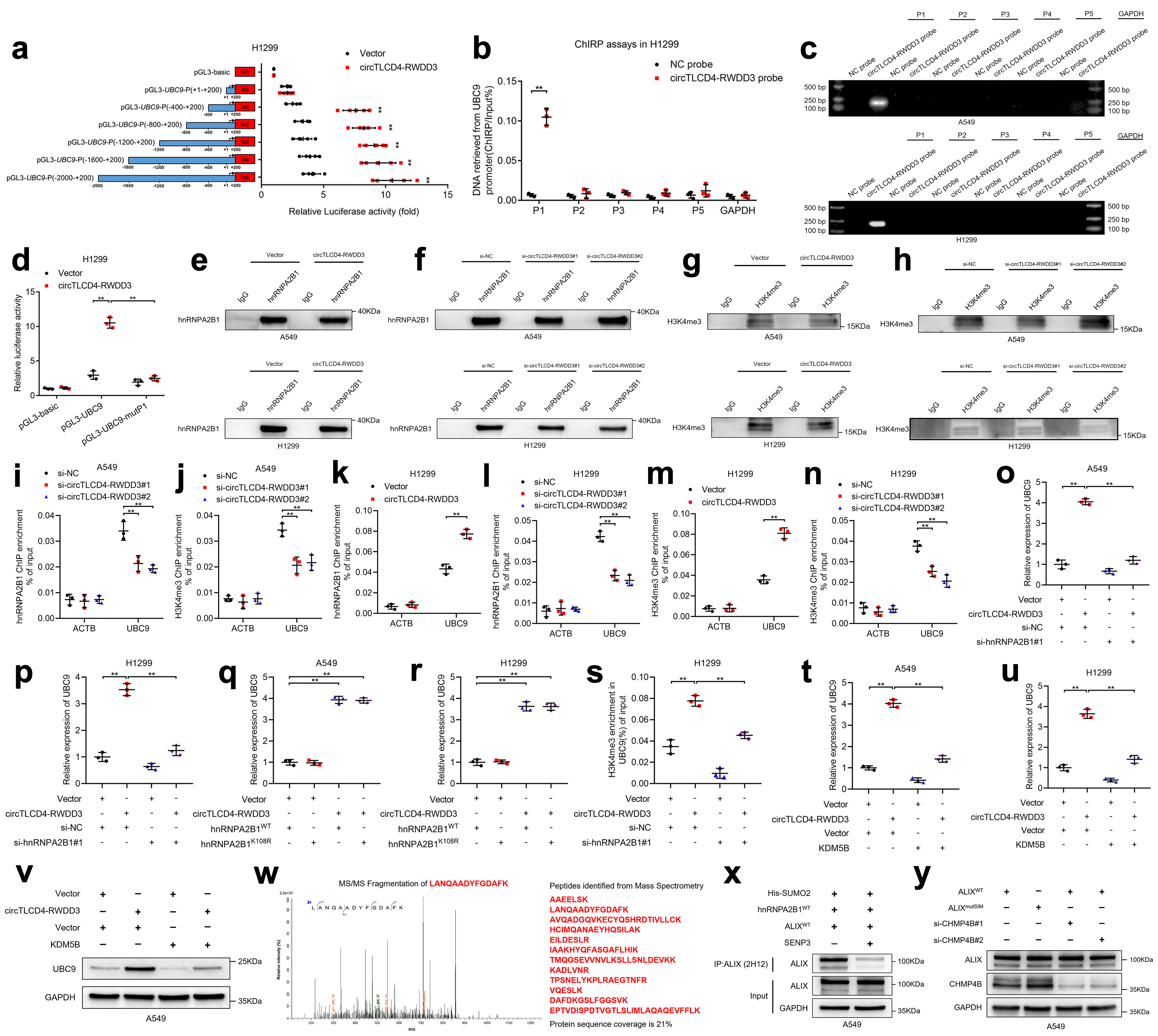


**Supplementary Figure S4. circTLCD4-RWDD3 enhances H3K4me3 modification by recruiting hnRNPA2B1 on *UBC9*** **promoter.**

**(a)** Transcriptional activity of *UBC9* in circTLCD4-RWDD3-overexpressing H1299 cells transfected with truncated *UBC9* promoter luciferase plasmids. **(b)** ChIRP assays to detect the circTLCD4-RWDD3-associated chromatin fragments of *UBC9* promoter in H1299 cells. **(c)** PCR analysis to show the specific fragment of *UBC9* promoter binding with circTLCD4-RWDD3. **(d)** Luciferase activity detected in H1299 cells after mutating the circTLCD4-RWDD3-binding site on *UBC9* promoter. **(e)** Western blotting analysis to show the IP efficiency of anti-hnRNPA2B1 antibodies in ChIP assays from A549 and H1299 cells with or without circTLCD4-RWDD3 overexpression. **(f)** Western blotting analysis to show the IP efficiency of anti-hnRNPA2B1 antibodies in ChIP assays from A549 and H1299 cells with or without knocking down circTLCD4-RWDD3. **(g)** Western blotting analysis to show the IP efficiency of anti-H3K4me3 antibodies in ChIP assays from A549 and H1299 cells with or without circTLCD4-RWDD3 overexpression. **(h)** Western blotting analysis to show the IP efficiency of anti-H3K4me3 antibodies in ChIP assays from A549 and H1299 cells with or without knocking down circTLCD4-RWDD3. **(i-j)** ChIP-qPCR assays to detect hnRNPA2B1 **(i)** and H3K4me3 **(j)** enrichment on *UBC9* promoter in circTLCD4-RWDD3-silencing A549 cells. **(k-l)** ChIP-qPCR assays to detect hnRNPA2B1 enrichment on *UBC9* promoter in circTLCD4-RWDD3-overexpressing **(k)** or circTLCD4-RWDD3-silencing H1299 cells **(l)**. **(m-n)** ChIP-qPCR assays to detect H3K4me3 enrichment on *UBC9* promoter in circTLCD4-RWDD3-overexpressing **(m)** or circTLCD4-RWDD3-silencing H1299 cells **(n)**. **(o-p)** qRT-PCR analysis of UBC9 expression in circTLCD4-RWDD3-overexpressing A549 **(o)** or H1299 cells **(p)** with or without knocking down hnRNPA2B1. **(q-r)** qRT-PCR analysis of UBC9 expression in circTLCD4-RWDD3-overexpressing A549 **(q)** or H1299 cells **(r)** with or without mutating K108 residue of hnRNPA2B1. **(s)** ChIP-qPCR assays to detect H3K4me3 enrichment on *UBC9* promoter in circTLCD4-RWDD3-overexpressing H1299 cells with or without knocking down hnRNPA2B1. **(t-u)** qRT-PCR analysis of UBC9 expression in circTLCD4-RWDD3-overexpressing A549 **(t)** and H1299 cells **(u)** with or without overexpressing KDM5B. **(v)** Western blotting analysis of UBC9 expression in circTLCD4-RWDD3-overexpressing A549 cells with or without overexpressing KDM5B. **(w)** Mass spectrometry analysis of SUMOylated hnRNPA2B1-interacting proteins after co-IP assay. **(x)** Western blotting analysis after co-IP assays to assess the activation of ALIX in A549 cells with or without SENP3 overexpression. **(y)** Western blotting analysis to show knockdown and overexpression efficiency in ALIX-overexpressing A549 cells with or without knocking down CHMP4B. The statistical difference was assessed with unpaired Student’s *t*-test in **b**, **k**, **m**, and **f**; and one-way ANOVA followed by Dunnett tests in **c**, **i-j**, **l**, **n-s**, and **u-v**. Error bars show the SD from three independent experiments. ^*^, *P* < 0.05; ^**^, *P* < 0.01.


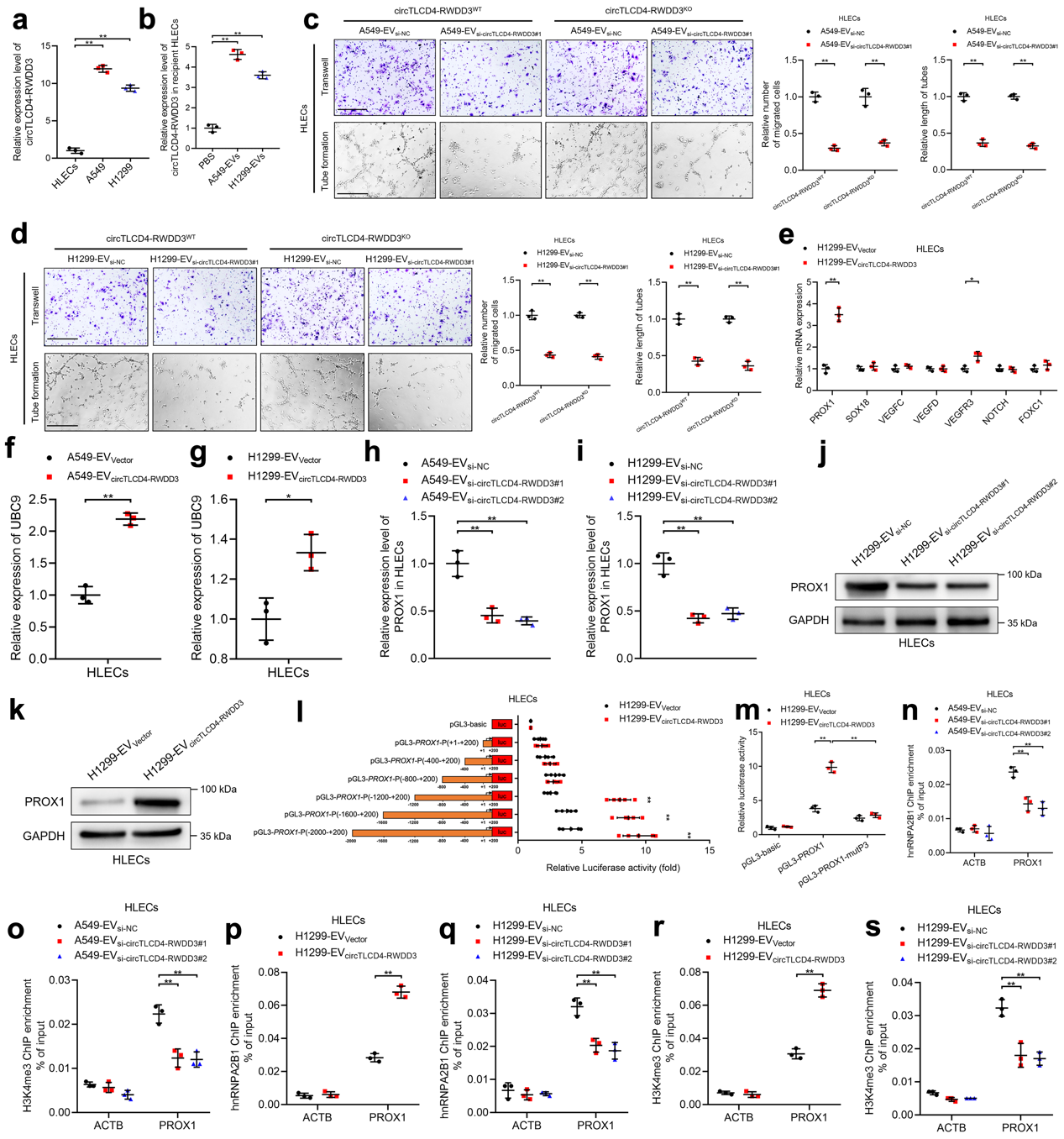


**Supplementary Figure S5. EV-packaged circTLCD4-RWDD3 upregulates PROX1 expression to facilitate lymphangiogenesis.**

**(a)** qRT-PCR analysis to show endogenous circTLCD4-RWDD3 expression in HLECs and NSCLC cell lines. **(b)** qRT-PCR analysis of the expression of circTLCD4-RWDD3 in HLECs treated with NSCLC cell-derived EVs or PBS. **(c)** Representative images and quantiﬁcation of the Transwell migration and tube formation of circTLCD4-RWDD3^WT^ and circTLCD4-RWDD3^KO^ HLECs treated with A549-EV_si-NC_ or A549-EV_si-circTLCD4-RWDD3#1_. Scale bars, 100μm. **(d)** Representative images and quantiﬁcation of the Transwell migration and tube formation of circTLCD4-RWDD3^WT^ and circTLCD4-RWDD3^KO^ HLECs treated with H1299-EV_si-NC_ or H1299-EV_si-circTLCD4-RWDD3#1_. Scale bars, 100μm. **(e)** qRT-PCR analysis of lymphangiogenesis-related gene expression in HLECs treated with H1299-EV_Vector_ or H1299-EV_circTLCD4-RWDD3_. **(f-g)** qRT-PCR analysis of UBC9 expression in HLECs treated with EV_Vector_ or EV_circTLCD4-RWDD3_ derived from A549 **(f)** or H1299 cells **(g)**. **(h-i)** qRT-PCR analysis of PROX1 expression in HLECs treated with EV_si-NC_, EV_si-circTLCD4-RWDD3#1_ and EV_si-circTLCD4-RWDD3#2_ derived from A549 **(h)** or H1299 cells **(i)**. **(j-k)** Western blotting analysis of PROX1 expression in HLECs treated with EVs derived from circTLCD4-RWDD3-silencing **(j)** or circTLCD4-RWDD3-overexpressing H1299 cells **(k)**. **(l)** Transcriptional activity of *PROX1* in H1299-EV_circTLCD4-RWDD3_-treated HLECs transfected with truncated *PROX1* promoter luciferase plasmids. **(m)** Luciferase activity detected in H1299-EV_Vector_- or H1299-EV_circTLCD4-RWDD3_-treated HLECs with or without mutating the circTLCD4-RWDD3-binding site on *PROX1* promoter. **(n-o)** ChIP-qPCR of the enrichment of hnRNPA2B1 **(n)** and H3K4me3 **(o)** on *PROX1* promoter in HLECs treated with EVs derived from A549 cells with or without silencing circTLCD4-RWDD3. **(p-q)** ChIP-qPCR of the enrichment of hnRNPA2B1 on *PROX1* promoter in HLECs treated with EVs derived from circTLCD4-RWDD3-overexpressing **(p)** or circTLCD4-RWDD3-silencing H1299 cells **(q)**. **(r-s)** ChIP-qPCR of the enrichment of H3K4me3 on *PROX1* promoter in HLECs treated with EVs derived from circTLCD4-RWDD3-overexpressing **(r)** or circTLCD4-RWDD3-silencing H1299 cells **(s)**. The statistical difference was assessed with one-way ANOVA followed by Dunnett tests in **a-b**, **h-i**, **m-o**, **q**, and **s**; and unpaired Student’s *t*-test in **c-g**, **p**, and **r**. Error bars show the SD from three independent experiments. ^*^, *P* < 0.05; ^**^, *P* < 0.01.


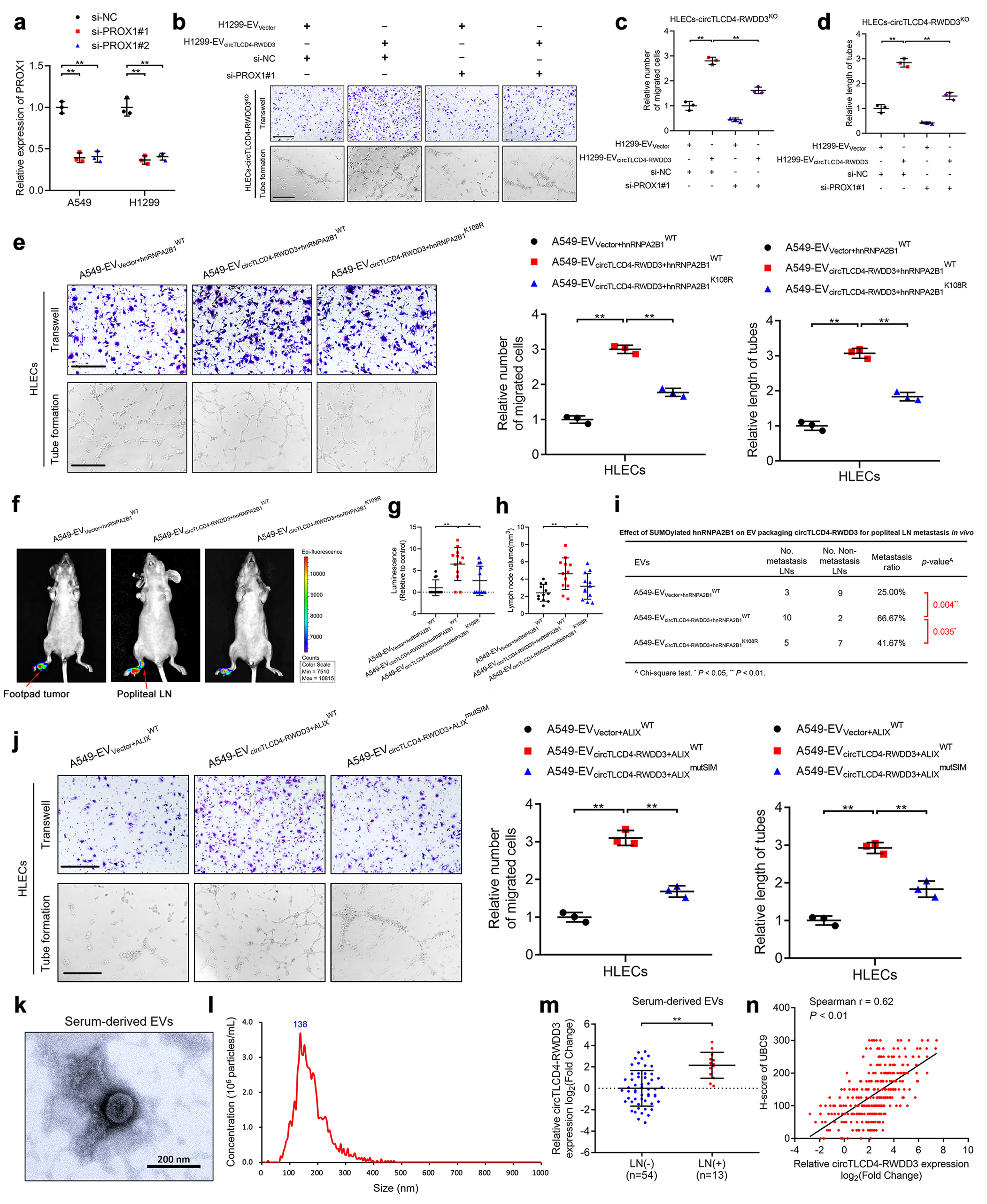


**Supplementary Figure S6. Blocking the transmission of EV-packaged circTLCD4-RWDD3** **inhibits lymphangiogenesis of NSCLC.**

**(a)** qRT-PCR analysis to show knockdown efficiency of PROX1 in A549 or H1299 cells. **(b-d)** Representative images **(b)** and quantification of Transwell migration **(c)** and tube formation **(d)** of H1299-EV_Vector_- or H1299-EV_circTLCD4-RWDD3_-treated circTLCD4-RWDD3^KO^ HLECs with or without knocking down PROX1. Scale bars, 100 µm. **(e)** Representative images and quantification of Transwell migration and tube formation of HLECs treated with EVs secreted by control or circTLCD4-RWDD3-overexpressing A549 cells with or without mutating K108 residue of hnRNPA2B1. Scale bars, 100 μm. **(f-g)** Representative images **(f)** and quantification of bioluminescence **(g)** of popliteal metastatic LNs from nude mice treated with EVs secreted by control or circTLCD4-RWDD3-overexpressing A549 cells with or without mutating K108 residue of hnRNPA2B1 (*n* = 12 per group). **(h)** Quantification of popliteal LN volume of nude mice treated with indicated EVs (*n* = 12 per group). **(i)** The percentage of metastatic popliteal LN in the nude mice (*n* = 12 per group). **(j)** Representative images and quantification of Transwell migration and tube formation of HLECs treated with EVs secreted by control or circTLCD4-RWDD3-overexpressing A549 cells with or without mutating SIM in ALIX. Scale bars, 100 μm. **(k-l)** TEM **(k)** and NTA **(l)** identified the characteristics of serum-derived EVs. Scale bars, 200 nm. **(m)** qRT-PCR analysis of circTLCD4-RWDD3 expression in serum-derived EVs from LN-positive and LN-negative NSCLC patients. **(n)** Correlation analysis of circTLCD4-RWDD3 and UBC9 expression in NSCLC tissues. The statistical difference was assessed with one-way ANOVA followed by Dunnett tests in **a**, **c-d**, **e**, **g**, **h**, and **j**; and Chi-square test in **i**; and nonparametric Mann-Whitney *U* test in **m**. Error bars show the SD from three independent experiments. ^*^, *P* < 0.05; ^**^, *P* < 0.01.


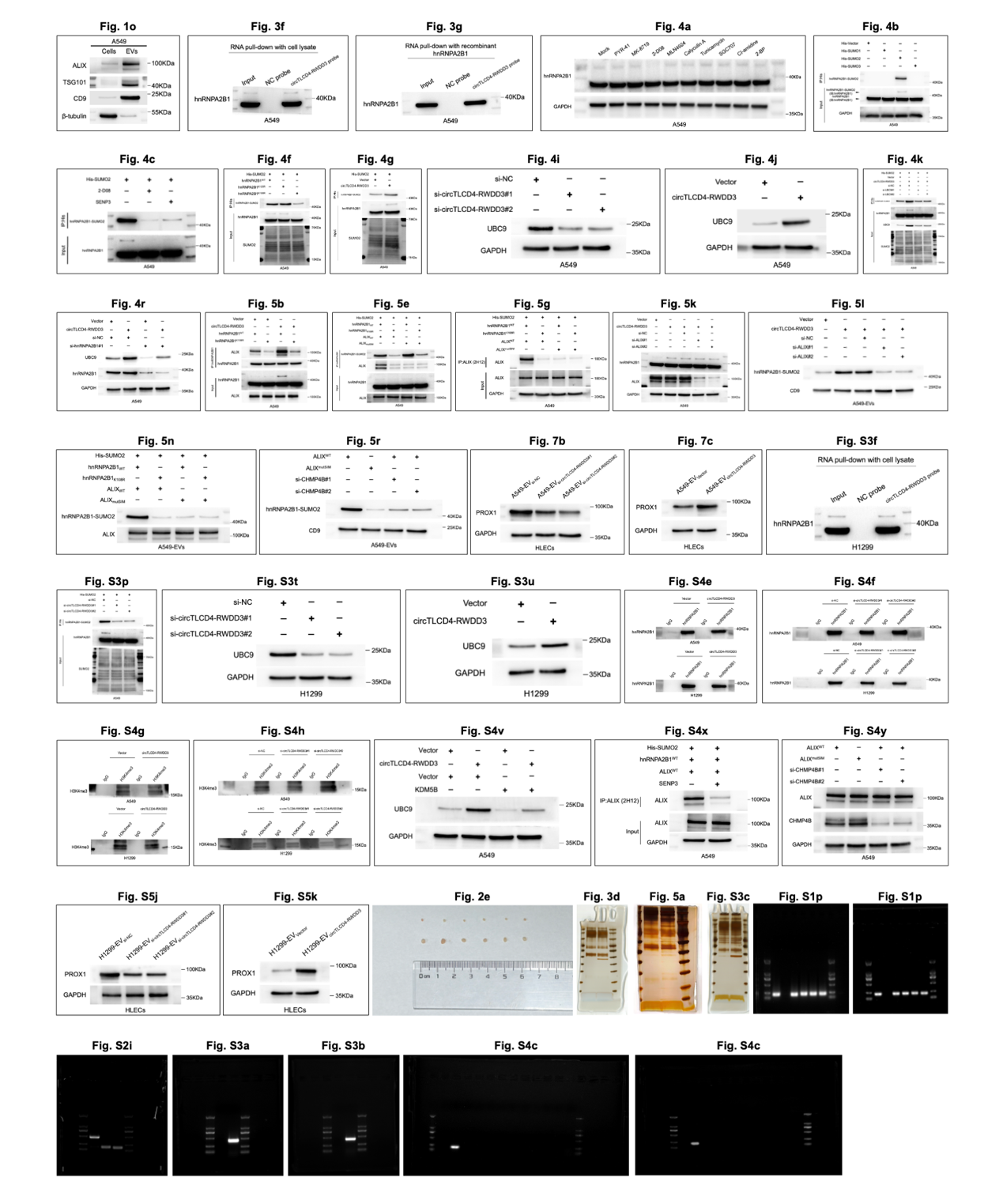


**Supplementary Figure S7. Full uncut original pictures.**

**Supplementary Table S1. Correlation between** **circTLCD4-RWDD3 expression and clinicopathological characteristics of** **NSCLC patients in multicenter cohort (n = 312).**

| **Characteristics** | **No. of cases** | **circTLCD4-RWDD3 expression** | | |
| --- | --- | --- | --- | --- |
|  |  | **Low** | **High** | ***P*-value^i^** |
| **Total cases** | 312 | 156 | 156 |  |
| **Sex** |  |  |  | 0.910 |
| male | 165 | 83 | 82 |  |
| female | 147 | 73 | 74 |  |
| **Age, yr** |  |  |  | 0.113 |
| < 60 | 150 | 68 | 82 |  |
| ≥ 60 | 162 | 88 | 74 |  |
| **TNM stage** |  |  |  | **0.001^**^** |
| I‑II | 188 | 110 | 78 |  |
| III‑IV | 124 | 46 | 78 |  |
| **Histological type** |  |  |  | 0.317 |
| adenocarcinoma | 245 | 117 | 128 |  |
| squamous cell carcinoma | 55 | 32 | 23 |  |
| large cell carcinoma | 12 | 7 | 5 |  |
| **Lymph node metastasis** |  |  |  | **0.001^**^** |
| positive | 87 | 18 | 69 |  |
| negative | 225 | 138 | 87 |  |

Abbreviations: No. of cases = number of cases. ^i^ Chi-square test, ^*^ *P* < 0.05, ^**^ *P* < 0.01.

**Supplementary Table S2. Univariate and multivariate Cox regression analysis of overall survival for circTLCD4-RWDD3 expression in multicenter NSCLC cohort (*n* = 312).**

| **Variables** | **Univariate analysis** | | | **Multivariate analysis** | | | |
| --- | --- | --- | --- | --- | --- | --- | --- |
|  | **HR** | **95%CI** | ***P*-value^i^** | | **HR** | **95%CI** | ***P*-value^i^** |
| Age (≥ 60 vs. < 60 yr) | 1.227 | 0.844-1.783 | 0.284 | |  |  |  |
| Sex  (male vs. female) | 1.067 | 0.726-1.569 | 0.741 | |  |  |  |
| TNM stage  (III‑IV vs. I‑II) | 1.380 | 0.917-2.075 | 0.122 | |  |  |  |
| Lymph node metastasis  (positive vs. negative) | 2.815 | 1.783-4.443 | **0.001^**^** | | 3.113 | 2.040-4.752 | **0.001****^**^** |
| circTLCD4-RWDD3 expression  (high vs. low) | 1.844 | 1.206-2.819 | **0.005^**^** | | 1.791 | 1.176-2.727 | **0.007^**^** |

Abbreviations: HR = hazard ratio; 95%CI = 95% confidence interval. **^i^** Cox regression analysis, ^*^ *P* < 0.05, ^**^ *P* < 0.01.

**Supplementary Table S3. Univariate and multivariate Cox regression analysis of disease-free survival for circTLCD4-RWDD3 expression in multicenter NSCLC cohort (*n* = 312).**

| **Variables** | **Univariate analysis** | | | **Multivariate analysis** | | | |
| --- | --- | --- | --- | --- | --- | --- | --- |
|  | **HR** | **95%CI** | ***P*-value^i^** | | **HR** | **95%CI** | ***P*-value^i^** |
| Age (≥ 60 vs. < 60 yr) | 1.072 | 0.757-1.516 | 0.696 | |  |  |  |
| Sex  (male vs. female) | 1.073 | 0.751-1.533 | 0.700 | |  |  |  |
| TNM stage  (III‑IV vs. I‑II) | 1.268 | 0.870-1.850 | 0.217 | |  |  |  |
| Lymph node metastasis  (positive vs. negative) | 2.121 | 1.387-3.244 | **0.001^**^** | | 2.314 | 1.555-3.443 | **0.001^**^** |
| circTLCD4-RWDD3 expression  (high vs. low) | 1.967 | 1.333-2.902 | **0.001^**^** | | 1.923 | 1.308-2.827 | **0.001^**^** |

Abbreviations: HR = hazard ratio; 95%CI = 95% confidence interval. **^i^** Cox regression analysis, ^*^ *P* < 0.05, ^**^ *P* < 0.01.

**Supplementary Table S4. The most abundant proteins identified by mass spectrometry after** **RNA pull-down analysis with** **circTLCD4-RWDD3 probes.**

| Protein | Peptides | Sequence coverage (%) |
| --- | --- | --- |
| hnRNPA2B1 | 8 | 35 |
| YBX1 | 7 | 36.8 |
| PAWR | 6 | 35.2 |
| PCBP1 | 7 | 29.8 |
| IGF2BP1 | 5 | 13.5 |
| C1QBP | 6 | 31.2 |
| ACTG1 | 6 | 29.4 |
| LIMCH1 | 7 | 14.2 |
| RPL34 | 3 | 24.1 |
| ITGB4 | 5 | 4.2 |

**Supplementary Table S5. The most abundant proteins identified by mass spectrometry after co-immunoprecipitation assay with hnRNPA2B1.**

| Protein | Peptides | Sequence coverage (%) |
| --- | --- | --- |
| SPATA7 | 10 | 19.8 |
| ALIX | 12 | 21 |
| EIF4G2 | 4 | 7.2 |
| DHX36 | 4 | 5.2 |
| THRAP3 | 3 | 3.1 |
| COPB2 | 5 | 7.2 |
| ESYT2 | 3 | 4.6 |
| SRRM1 | 2 | 5.5 |
| SMARCAL1 | 5 | 8 |
| HKDC1 | 1 | 2.8 |

**Supplementary Table S6. Correlation between circTLCD4-RWDD3 expression in serum-derived EVs and clinicopathological characteristics of NSCLC patients (n = 67).**

| **Characteristics** | **No. of cases** | **circTLCD4-RWDD3 expression**  **in** **serum-derived EVs** | | |
| --- | --- | --- | --- | --- |
|  |  | **Low** | **High** | ***P*-value^i^** |
| **Total cases** | 67 | 34 | 33 |  |
| **Sex** |  |  |  | 0.710 |
| male | 35 | 17 | 18 |  |
| female | 32 | 17 | 15 |  |
| **Age, yr** |  |  |  | 0.180 |
| < 60 | 38 | 22 | 16 |  |
| ≥ 60 | 29 | 12 | 17 |  |
| **TNM stage** |  |  |  | **0.019^*^** |
| I‑II | 40 | 25 | 15 |  |
| III‑IV | 27 | 9 | 18 |  |
| **Histological type** |  |  |  | 0.582 |
| adenocarcinoma | 53 | 27 | 26 |  |
| squamous cell carcinoma | 13 | 7 | 6 |  |
| large cell carcinoma | 1 | 0 | 1 |  |
| **Lymph node metastasis** |  |  |  | **0.005^**^** |
| positive | 13 | 2 | 11 |  |
| negative | 54 | 32 | 22 |  |

Abbreviations: No. of cases = number of cases. ^i^ Chi-square test, ^*^ *P* < 0.05, ^**^ *P* < 0.01.

**Supplementary Table S7. Primers and probes used in the experiments.**

| **Target gene** | **Sequence (5’-3’)** | **Application** |
| --- | --- | --- |
| circTLCD4-RWDD3 | F: AGGCTACCTCATTTCTGATTTGT  R: GGAGAAACTTTTGCTGAAAACCA | qRT-PCR |
| TLCD4-RWDD3 | F: GAGTGCTGGCATACATTGGG  R: AACACGGTCCCATCTGTCTT | qRT-PCR |
| U1 | F: CAGGGGAGATAACGTGACCA  R: GGGAAAAGCACGGACACAG | qRT-PCR  RIP |
| 18S rRNA | F: AGCAGACATTGACCTCACCA  R: CCTCTATGGGCCCGAATCTT | qRT-PCR |
| GAPDH | F: CAAATTCCATGGCACCGTCA  R: ATGATGTTCTGGAGAGCCCC | qRT-PCR  ChIRP |
| TSG101 | F: GGCTACTGGACACATACCCA  R: GGACGAGAGAAGACTGGAGG | qRT-PCR |
| SAE1 | F: GGTGGCTGTCTTTGTTCCAG  R: AGAAGGTGACAAGAGGCTCC | qRT-PCR |
| UBA2 | F: GCTGCCCGAAACCATGTTAA  R: AGGAAAGGTTCTCTGGGTCG | qRT-PCR |
| UBC9 | F: AATTCTTCGTCCTGAGGCCA  R: GCTTCCCATCTCTGTCCACT | qRT-PCR |
| SENP1 | F: ACAGCCAGAGACCTTGGAAA  R: GATGTCCCTCACCCCTTTCA | qRT-PCR |
| SENP2 | F: TGGCTGGTTAGGATTCTCGG  R: TGGTCAGCTGGAATGGGAAT | qRT-PCR |
| SENP3 | F: AAATGAATGTGGCCAGGCAG  R: TGAGTTTGCAGTGACACAGC | qRT-PCR |
| SENP5 | F: CAGTTGCAGCCCATTTCCTT  R: GTTGGTCAGGGAATGCTTCG | qRT-PCR |
| SENP6 | F: TGGAAAAGTAGAAGCAGCGC  R: ACGACGTTTCAGAGGTGTGT | qRT-PCR |
| SENP7 | F: TCGTCTCACTGGTATCTCGC  R: TGGGAATCCTCTGCACTCAA | qRT-PCR |
| UBC9-P1 | F: GACCCGGTCCTGCTTGTT  R: GGAGGAGAAGTGTGGATGCT | ChIRP |
| UBC9-P2 | F: AATGAGTGAGGGAGGGAATG  R: CTCCCCTACTCCGTCATTCC | ChIRP |
| UBC9-P3 | F: ATGAGTTTGTGAGAGTGGGA  R: CTCACTCTCACTCATTCCCG | ChIRP |
| UBC9-P4 | F: GCCCCTGCTGCCCTGGACCC  R: AAACTCATTCCCTCCCTCACT | ChIRP |
| UBC9-P5 | F: AAGCAGATTCCCAGGACCTC  R: CCCCAGAATCCAGTCCCTG | ChIRP |
| ACTB | F: TGACAAAACCTAACTTGCGCA  R: CAATCAAAGTCCTCGGCCAC | ChIP |
| gRNA-TLCD4-RWDD3-Alu | F: GGGCTTAAATACCTGCTACA  R: TGCCAGCCAAGTCTTCCTTT | qRT-PCR |
| PROX1 | F: CAGCCCGAAAAGAACAGAAG  R: GGGTCTAGCTCGCACATCTC  GGGTCTAGCTCGCACATCTC  CAGCCCGAAAAGAACAGAAG | qRT-PCR |
| SOX18 | F: AGAAGCGTCACTGTGGCAAA  R: TTATTGTGGCCTCTCCGTCC | qRT-PCR |
| VEGF-C | F: TGGGGAAGGAGTTTGGAGTC  R: GTTACTGGTTTGGGGCCTTG | qRT-PCR |
| VEGF-D | F: ACCTTCCATTCACACCAGCT  R: GACACCTGCCATTCCATGAC | qRT-PCR |
| VEGFR3 | F: AGGAGAAAGAGCCACATCCC  R: ATATCCTGGAGTAACGCGCA | qRT-PCR |
| NOTCH | F: GACCTCATCAACTCACACGC  R: GGTGTCTCCTCCCTGTTGTT | qRT-PCR |
| FOXC1 | F: TCTTCCTTGCCTCTCACCTG  R: ACACTTTCTGGCGTTTGGTC | qRT-PCR |
| PROX1-P1 | F: CCCCCACCCCTTTTATATTT  R: ACAGGAAGACTGCACGTCAC | ChIRP |
| PROX1-P2 | F: GCGTCCTGGAAGAGCTAGTG  R: GGAAGAGAGGAGGGGAGAGG | ChIRP |
| PROX1-P3 | F: TTAGGTCATAAACGCGGCTG  R: CGCATAAATCGCAGGCTCTA | ChIRP |
| PROX1-P4 | F: CTGCGATTTATGCGTTTGAA  R: TTGCAAACATCTGGCGATTA | ChIRP |
| PROX1-P5 | F: TTGAGGGACACACGAAGACT  R: CTCCTTGGGCTTTGCAAGTT | ChIRP |
| circTLCD4-RWDD3-gRNA1 | TAGTGGCCCACAATCTAGGC CGG | CRISPR/Cas9 |
| circTLCD4-RWDD3-gRNA2 | CAAGACTCCATCTGGAGGCG GGG | CRISPR/Cas9 |
| si-circTLCD4-RWDD3#1 | sense: ACCUUGUACUGGUUGAAGAAATT  antisense: UUUCUUCAACCAGUACAAGGUTT | si-RNA |
| si-circTLCD4-RWDD3#2 | sense: UACCUUGUACUGGUUGAAGAATT  antisense: UUCUUCAACCAGUACAAGGUATT | si-RNA |
| si-UBC9#1 | sense: GGGAUUGGUUUGGCAAGAATT  antisense: UUCUUGCCAAACCAAUCCCTT | si-RNA |
| si-UBC9#2 | sense: CAAUGAACCUGAUGAACUGTT  antisense: CAGUUCAUCAGGUUCAUUGTT | si-RNA |
| si-hnRNPA2B1#1 | sense: GCAAUUCAUUGAGCGCAUUTT  antisense: GCAAUUCAUUGAGCGCAUUTT | si-RNA |
| si-hnRNPA2B1#2 | sense: GCUCUUUAUUGGUGGCUUATT  antisense: UAAGCCACCAAUAAAGAGCTT | si-RNA |
| si-ALIX#1 | sense: CCAGAACAAAUGCAGUGAUAUTT  antisense: AUAUCACUGCAUUUGUUCUGGTT | si-RNA |
| si-ALIX#2 | sense: CCUGAAUUACUGCAACGAAAUTT  antisense: AUUUCGUUGCAGUAAUUCAGGTT | si-RNA |
| si-CHMP4B#1 | sense: GUUAAGCAAGAAACAGGAGUUTT  antisense: AACUCCUGUUUCUUGCUUAACTT | si-RNA |
| si-CHMP4B#2 | sense: CGGCACAUUAUCAACCAUCGATT  antisense: UCGAUGGUUGAUAAUGUGCCGTT | si-RNA |
| si-PROX1#1 | sense: UGGAGAAGUAUGCGCGUCATT  antisense: UGACGCGCAUACUUCUCCATT | si-RNA |
| si-PROX1#2 | sense: UUUCCAGGAGCAACCAUAAUUTT  antisense: AAUUAUGGUUGCUCCUGGAAATT | si-RNA |
| circTLCD4-RWDD3 | CCATATTTCTTCAACCAGTACAAGGTAGTAT  5’-Cy3 labeled and 3’-Cy3 labeled | FISH |

**Supplementary Table S8. Antibodies used in the experiments.**

| **Product** | **Source** | **Catalog no.** |
| --- | --- | --- |
| **Primary antibody** |  |  |
| ***Western blotting*** |  |  |
| anti-hnRNPA2B1 | Cell Signaling Technology | 9304 |
| anti-ALIX | Cell Signaling Technology | 92880 |
| anti-TSG101 | Abcam | ab125011 |
| anti-CD9 | Cell Signaling Technology | 13403 |
| anti-SUMO2 | Abcam | ab233222 |
| anti-UBC9 | Abcam | ab75854 |
| anti-PROX1 | Abcam | ab199359 |
| anti-H3K4me3 | Abcam | ab8580 |
| anti-CHMP4B | Cell Signaling Technology | 35644 |
| anti-GAPDH | Abcam | ab8245 |
| ***Immunohistochemistry*** |  |  |
| anti-LYVE-1 | Abcam | ab218535 |
| anti-mCherry | Abcam | ab125096 |
| anti-PROX1 | Cell Signaling Technology | ab199359 |
| ***Immunofluorescence*** |  |  |
| anti-hnRNPA2B1 | Cell Signaling Technology | 9304 |
| anti-PROX1 | Abcam | ab199359 |
| anti-LYVE-1 | Abcam | ab218535 |
| anti-UBC9 | Abcam | ab75854 |
| anti-TGN46 | Abcam | ab16059 |
| anti-Rab5 | Abcam | ab218624 |
| anti-Rab7 | Abcam | ab137039 |
| anti-CD63 | Abcam | ab134045 |
| anti-ALIX | Cell Signaling Technology | 92880 |
| anti-CHMP4B | Proteintech | 13683-1-AP |
| Phalloidin-iFluor 488 | Abcam | ab176753 |
| ***Immunoprecipitation*** |  |  |
| anti-hnRNPA2B1 | Cell Signaling Technology | 9304 |
| anti-6X His tag | Abcam | ab18184 |
| anti-Flag tag | Cell Signaling Technology | 14793 |
| anti-ALIX(2H12) | Santa Cruz | sc-53539 |
| anti-H3K4me3 | Abcam | ab8580 |
| **Secondary antibody:** |  |  |
| ***Western blotting*** |  |  |
| anti-rabbit IgG-HRP | Cell Signaling Technology | 7074 |
| anti-mouse IgG-HRP | Cell Signaling Technology | 7076 |
| ***Immunohistochemistry*** |  |  |
| anti-rabbit IgG-HRP | Proteintech | SA00001-2 |
| anti-mouse IgG-HRP | Proteintech | SA00001-1 |
| ***Immunofluorescence*** |  |  |
| [Alexa Fluor 594](https://www.baidu.com/link?url=nF9d2Xaur7vyZuSh6bwYgXJHxCoqgi5ljmVkB6q--I4j4E8mmfQwWu1WHii3mT9LmMQ5XQE23xsmXGJKAUgtmctb7vuX9L1odcCwYmhMNRgtKDKfaFBBlPGw7dU_i5LpQii0iVI7_AAuccqxBIw54_&wd=&eqid=cad6a7aa0006a5040000000661054afd) | Abcam | ab150080/ab150116 |
| [Alexa Fluor 4](https://www.baidu.com/link?url=nF9d2Xaur7vyZuSh6bwYgXJHxCoqgi5ljmVkB6q--I4j4E8mmfQwWu1WHii3mT9LmMQ5XQE23xsmXGJKAUgtmctb7vuX9L1odcCwYmhMNRgtKDKfaFBBlPGw7dU_i5LpQii0iVI7_AAuccqxBIw54_&wd=&eqid=cad6a7aa0006a5040000000661054afd)88 | Abcam | ab150077/ab150113 |

**Supplementary materials and methods**

***Tube formation assay***

A mixture of Matrigel (BD Biosciences, Franklin Lakes, NJ, USA, catalog no. 356234) and ECM in a 1:2 ratio (v/v) was added to 24-well plates (400 µl per well), incubated at 37 °C overnight, then HLECs (1 × 10^5^/well) treated with 10 μg/ml EVs derived from different groups of transfected cells were seeded into Matrigel-coated plates prior to 3-5 hour incubation. The formation of lymphatic vessels was imaged using an Olympus IX73 (Tokyo, Japan), and the tube formation ability was determined by measuring the number of tube junctions and branch length using Image J software (US National Institutes of Health, Bethesda, MD, USA).

***Transwell assay***

Based on the manufacturer’s protocol, the migration ability of HLECs were evaluated by Transwell assays using Transwell chambers (Corning Costar Corp, USA, catalog no. 3422). HLECs (1 × 10^5^/well) previously treated with 10 μg/ml EVs derived from different groups of transfected cells were resuspended in 300 μL serum-free medium and added into the upper chamber, while 700 μL of serum-containing culture medium was added to the lower chamber. After incubation for 3-5 hours, the chambers were washed three times using phosphate-buffered saline (PBS). Cells that migrated into the lower chamber were fixed in 4% paraformaldehyde and stained for 20 min with 0.1% crystal violet. Images were captured using an Eclipse 80i microscope (Nikon, Tokyo, Japan) and analyzed using Image J software. The number of migrated cells was averaged over five fields of view per condition.

***Immunohistochemistry (IHC) analysis***

For IHC analysis, formalin-ﬁxed, parafﬁn-embedded tissues were sectioned at 4 μm thickness. Then, the sections were dewaxed in xylene, rehydrated in alcohol, retrieved in EDTA buffer (pH 8.0) and blocked by peroxidase inhibitors. Specimens were incubated with normal goat serum for 30 min at room temperature to block non-speciﬁc binding. Then sections were incubated overnight at 4 °C with antibodies, rinsed with PBS, and incubated with biotinylated secondary antibodies for 30 min at room temperature, followed by color development through 3,3'-Diaminobenzidine (DAB) and hematoxylin. Finally, the Eclipse 80i microscope (Nikon) was applied for the capture of images.

***Fluorescence* in situ *hybridization (FISH) assay***

FISH assay was performed on cell cultures using the Ribo FISH Kit (RiboBio, Guangzhou, China, catalog no. C10910). Briefly, 2 × 10^4^ NSCLC cells were seeded on a confocal dish, incubated for 24 h, fixed using 4% paraformaldehyde at room temperature for 10 min, treated with 0.5% Triton X-100 for another 10 min, then incubated in prehybridization solution at 37 °C for 30 min. After incubating in prehybridization solution at 37°C for 30 min, Cy3-labeled circTLCD4-RWDD3 probe (GenePharma, Suzhou, China) was hybridized with cells at 37°C overnight. Dishes were washed sequentially with 4 ×, 2 × and 1 × saline sodium citrate buffer; counterstained with DAPI; and imaged under an LSM710 confocal microscope (Carl Zeiss, Oberkochen, Germany).

FISH assay was performed on NSCLC tissues using an RNA FISH kit (GenePharma, catalog no. F22201/50) according to the manufacturer’s protocol. Sections were digested with proteinase K and hybridized at 37 °C overnight with Cy3-labeled probes against circTLCD4-RWDD3 (GenePharma). Nuclei were counterstained with DAPI. Images were captured using an LSM710 confocal microscope (Carl Zeiss).

***Immunofluorescence***

NSCLC cells (2 × 10^4^) were cultured in confocal dishes, fixed using 4% paraformaldehyde for 10 min, permeabilized using 0.5% Triton X-100 for 15 min, blocked with 1% BSA at 37 °C for 30 min, then incubated with primary antibodies at 4 °C overnight, followed by appropriate corresponding secondary antibodies for 1 h. Cultures were counterstained with DAPI for 15 min. Images were captured using an LSM710 confocal microscope (Carl Zeiss).

NSCLC tissues were incubated with primary antibodies at 37 °C for 1 h, followed by fluorophore-conjugated secondary antibodies at room temperature for 30 min. Nuclei were stained with DAPI. Images were captured using an LSM710 confocal microscope (Carl Zeiss).

***Histology evaluation of tissue sections***

As for the evaluation of circTLCD4-RWDD3, the percentage of positive staining tumor cells was designated follows: 0 (no positive), 1 (0-10% positive), 2 (10-30% positive), 3 (30–70% positive), and 4 (over 70% positive). The staining intensity was graded as follows: 1 (no staining), 2 (weak staining), 3 (moderate staining) and 4 (strong staining). The staining index (SI) was calculated by multiplying positive percentage with staining intensity with possible scores of 0, 1, 2, 3, 4, 6, 8, 9, 12, and 16. Then, the median value, which is SI = 8, was defined as the cut off value. And samples with a SI ≥ 8 were defined as high expression and samples with a SI < 8 were low expression. As for the quantification of LYVE-1, the number of positive staining of vessels was calculated by Image J software (NIH) in three random fields for each section. And the median value was defined as the cut off value.

***Subcellular fraction assay***

To identify the subcellular location of circTLCD4-RWDD3, subcellular fraction assays were conducted according to the manufacturer’s instructions of the PARIS^TM^ kit (Thermo Scientific, Waltham, MA, USA, catalog no. AM1921). Briefly, 1 × 10^6^ NSCLC cells were harvested and lysed in ice-cold Cell Fractionation Buffer for 5 min. After centrifugation at 500 g for 5 min at 4°C to separate the cytoplasm and nucleus, RNA from nuclear and cytoplasm was extracted using Trizol reagents (Takara Bio, Japan, catalog no. 9109). qRT-PCR analysis was performed to determine the subcellular location of circTLCD4-RWDD3. 18S RNA and U1 were used as internal references.

***Isolation and purification of EVs***

To isolate EVs from NSCLC cells, the cells were cultured for 48 h in medium containing 10% EV-depleted FBS. Then, the samples were centrifuged sequentially at 2,000 *g* for 10 min, 10,000 *g* for 30 min, and 120,000 *g* for 70 min at 4°C to obtain purified EVs. The purified EVs were resuspended in PBS and stored at -80 ℃ until further analysis.

To isolate EVs from NSCLC and adjacent NATs, samples were washed with ice-cold PBS, sliced, and digested into a single-cell suspension using the Tumor Dissociation Kit (Miltenyi Biotec, Germany, catalog no. 130-093-237). The dissociated tissues were centrifuged at 500 *g* for 7 min at 4 °C, followed by centrifugation at 2,000 *g* for 10 min at 4°C to obtain cell-free supernatant. The resulting supernatant was processed through the sequential centrifugations as above in order to obtain EVs.

For the isolation of EVs from serum samples, 2ml fresh NSCLC patient blood was collected and allowed to clot for 1 hour at 37 ˚C without anticoagulation. Thereafter, the fresh blood was centrifuged at 2,000 *g* for 10 min to obtain serum, which was diluted in sterile PBS in a 1:1 ratio. Subsequently, the diluted serum was processed through the sequential centrifugations to obtain and purify EVs.

***Electron microscopy analysis***

Isolated EVs were deposited on grids for 60 min, fixed with 2.5% glutaraldehyde for 10 min, washed five times with PBS, incubated with uranyl acetate for 5 min, then washed again with PBS. Grids were imaged using a transmission electron microscope (Hitachi, Tokyo, Japan).

***Nanoparticle tracking analysis***

Isolated EVs were suspended in PBS to a final concentration up to 2 × 10^9^ particles/ml, then Nanosight LM10 (Malvern, Framingham, MA) was applied to assess the concentration and size of EVs by recording for 30 s with a 488 nm laser at 25°C. Analysis was conducted using the NTA v3.1 software (Malvern, Framingham, MA).

***Lentivirus infection and cell transfection***

To construct the circTLCD4-RWDD3 stable-overexpressing NSCLC cell lines, concerned lentivirus, prepared by Genepharma, were used to infect the NSCLC cells. Infected NSCLC cells were selected for 2 weeks using puromycin (Sigma-Aldrich, catalog no. 540411).

For cell transfection, 2 × 10^5^ cells per well were seeded into the 6-well plate and cultured overnight at 37°C in 5% CO_2_. The indicated plasmids and small interfering RNAs (siRNAs) (Genepharma) were transfected into cells with the presence of lipofectamine 3000 (Invitrogen, Carlsbad, CA, USA, catalog no. L3000008) according to the protocols from manufacturer. qRT-PCR analysis was used to check the transfection efficiency. The hnRNPA2B1^K108R^ or ALIX^mutSIM^ cell lines were constructed by transfecting with corresponding mutating plasmids in NSCLC cells as mentioned above.

***RNA extraction and qRT-PCR analysis***

Total RNA was extracted from cells, tissues, and EVs using TRIzol (Takara Bio) based on manufacturer's instructions, then reverse-transcribed into cDNA using the Hiscript III Reverse Transcriptase kit (Vazyme, Nanjing, China, catalog no. R312-01). Subsequently, corresponding RNA expression was evaluated by qRT-PCR analysis with ChamQTM Universal SYBR qPCR Master Mix kit (Vazyme, catalog no. Q711-02). The detailed sequences of primers are listed in **Supplementary Table 7**.

***Western blotting analysis***

Harvested cells were washed with PBS three times, then lysed with RIPA lysis buffer (Thermo Scientific, catalog no. 89900) supplemented with a cocktail of phosphatase inhibitors and protease inhibitors at 4°C for 30 min. The cell lysate was spun at 12,000 *g* for 30 min at 4°C, followed by collecting the supernatant and estimating total protein concentration using the BCA kit (CWBio, Taizhou, China, catalog no. CW0014). Subsequently, equal amounts of protein were separated by 10% SDS polyacrylamide gels (SDS-PAGE) gel electrophoresis and transferred onto a polyvinylidene fluoride (PVDF) membrane, which were blocked with 5% BSA for 1 h at room temperature, incubated at 4 °C overnight with primary antibodies, washed three times with Tris-buffered saline containing 0.1% Tween-20, then incubated with horseradish peroxidase-conjugated secondary antibodies for 1 h at room temperature. The immunoblots were detected by the ECL chemiluminescence kit (Thermo Scientific, catalog no. 32209) and quantified using Image J software (NIH).

***Purification of recombinant hnRNPA2B1 proteins***

Briefly, full-length hnRNPA2B1 was cloned into pET-28a(+) vector (Novagen, Darmstadt, Germany, catalog no. 69864) carrying an C-terminal His tag. The plasmids were transformed into *E. coli* BL21(DE3), inoculated in LB broth (containing kanamycin) and grown at 37 °C until an optical density at 600 nm of 0.7 was reached. Then, isopropyl-β-d-thiogalactopyranoside (to a final concentration of 0.4 mM) was added. After growing for 3 h at 37 °C, *E. coli* were lysed in buffer (20 mM sodium phosphate, pH 7.5, 10 mM imidazole, 0.5 M NaCl and EDTA-free protease inhibitors) and the mixture was sonicated. The His-tagged protein was isolated from the supernatants using a HisPur Ni-NTA Purification Kit (Thermo Scientific, catalog no. 88229).

***Mass spectrometry analysis***

The different bands from silver staining were subjected to the MALDI-TOF spectrometry instrument. Subsequently, serval peptides were identified according to the b, y ion signals of the tested proteins. Based on combined MS and tandem mass spectrometry (MS/MS spectra), the proteins were successfully identified based on 95% or higher confidence interval of their scores in the MASCOT V2.3 search engine (Matrix Science Ltd., London, U.K.), using the following search parameters, NCBInr-Aspergillus niger database.

***Inhibiting post-translational modification***

we have used various inhibitors to detect the potential post-translational modification of hnRNPA2B1, in which PYR-41 is a specific inhibitor for ubiquitylation, MK-8719 for O-GlcNAcylation, 2-D08 for SUMOylation, MLN4924 for NEDDylation, Calyculin A for phosphorylation, Tunicamycin for N-linked glycosylation, SGC707 for arginine methylation, CI-amidine for deimination and 2-BP for palmitoylation.

***Chromatin isolation by RNA purification (ChIRP) assay***

ChIRP assays were performed to confirm the interaction between circTLCD4-RWDD3 and chromatins using the Magna ChIRP RNA Interactome Kit (Millipore, catalog no. 17-10494) following the manufacturer’s instructions. Harvested NSCLC cells and HLECs were fixed in 1% formaldehyde and lysed in cell lysis buffer at 4°C for 10 min. Next, the chromatin in cell lysate was sheared into 500-700 bp fragments by sonication at 4°C for 1 h. The chromatin sample was incubated with biotinylated circTLCD4-RWDD3 probes (Genepharma) and recovered with streptavidin-conjugated magnetic beads at 4°C overnight. After washing up with washing buffer 5 times, the combined DNA was extracted for further qRT-PCR analysis.

***Chromatin immunoprecipitation (ChIP) assay***

The DNA fragments interacting with hnRNPA2B1 and H3K4me3 were examined by ChIP assays according to the manufacturer’s protocol of EZ-Magna ChIP A/G kit (Millipore, catalog no. 17-371). 1 × 10^7^ NSCLC cells or HLECs treated with indicated EVs were harvested and crosslinked in 4% paraformaldehyde at room temperature for 10 min, followed by isolating the chromatins with cell lysis buffer and nuclear lysis buffer. The chromatin was further sonicated to generate short fragments between 500 bp and 700 bp and then hybridized with specific antibodies. The binding complexes were immunoprecipitated with protein A/G-coated magnetic beads at 4°C overnight. The cross-linking DNA was eluted from the beads and evaluated by qRT-PCR analysis.

***Co-immunoprecipitation (co-IP) assay***

Co-IP assays were conducted using the co-Immunoprecipitation Kit (Thermo Fisher, catalog no. 26149). Briefly, NSCLC cells were lysed in the co-IP lysis buffer and centrifuged at 12,000 *g* for 30 min. The supernatant was incubated at 4 °C overnight with magnetic beads conjugated to the specific antibodies. Moreover, the normal mouse/rabbit immunoglobulin G (IgG) was used as a negative control. The proteins were eluted to be further analyzed by western blotting.

***RIP assay***

RIP assays were performed to confirm the interaction between circTLCD4-RWDD3 and hnRNPA2B1 using the EZ-Magna RIP kit (Merck, Darmstadt, Germany, catalog no. 17-701). Briefly, 2 × 10^7^ NSCLC cells were harvested and lysed in RIP lysis buffer at 4°C for 10 min. After storing the cell lysate at -80°C for at least 2 h, the supernatant of lysate was collected by centrifugation for 10 min at 12,000 *g*. Then, the supernatant was co-immunoprecipitated with magnetic beads conjugated with antibodies at 4°C overnight. The retrieved RNAs were eluted and further subjected to qRT-PCR analysis.

During the mutating assays, the circTLCD4-RWDD3 mutant with mutations of 340-390 nt or 375-378 nt were conducted by mutating A to U, C to G, U to A or G to C. And then, the NSCLC cells with circTLCD4-RWDD3 mutant were subjected into RIP assays as mentioned above.

***Silver staining***

After RNA pull-down assays and co-IP assays, equal volume of protein samples was electrophoretic separated by 10% SDS-PAGE gel, followed by washing and silver staining using the silver stain kit (Thermo Scientific, catalog no. 24612) according to manufacturer’s instruction.

***Dual-luciferase reporter assay***

The dual-luciferase reporter assays were conducted to detect the transcriptional regulation of circTLCD4-RWDD3 on the promoter of *UBC9* and *PROX1*. Briefly, serial truncations of the promoter regions of human *UBC9* and *PROX1* were inserted upstream of the promoter of the luciferase gene in pGL3-Basic luciferase reporter vector. Next, the constructed pGL3 plasmids were co-transfected into NSCLC cells or HLECs. After incubating for 48 hours, the cells were harvested and lysed, followed by measuring firefly and renilla luciferase activity using the Dual-Luciferase Reporter Assay System (Promega, Madison, WI, USA).

The sequence analysis to predict the specific interacting site of circTLCD4-RWDD3 on *UBC9* or *PROX1* promoter were conducted using the plug-in Megalign in DNAstar software. Then, the mutating dual-luciferase reporter assays were performed by mutating nucleotide A to T, T to A, C to G or G to C in the predicted regions of *UBC9* or *PROX1* promoter.

***Target DNA deletion by CRISPR/Cas9 technology***

Single guide RNAs (gRNAs) targeted the Alu element in the intron upstream of TLCD4-RWDD3 exon 2 were designed and inserted into LentiCRISPR v2 vectors (UBIgene, Guangzhou, China). The constructed plasmids were transfected into the HLECs to knockout the expression of circTLCD4-RWDD3. Genomic DNA was extracted after 72 h transfection using the Universal Genomic DNA Kit (CWBio, catalog no. CW2298). qRT-PCR analysis was conducted to determine the knockout efficiency.

***CircRNA sequencing***

Total RNA samples (2 μg) were extracted and reverse transcribed as the same method described in RNA extraction and qRT-PCR analysis. According to the manufacturer’s protocols, 2 × Es Taq MasterMix (Dye) kit (CWBio, catalog no. CW0690) was used to amplify the sequence of the back-splicing site in circTLCD4-RWDD3. Subsequently, sanger sequencing was performed to verify the specific sequence of the back-splicing site in circTLCD4-RWDD3.

***RNase R treatment and actinomycin D assay***

To examine the stability of circTLCD4-RWDD3, the RNase R treatment and actinomycin D assays were performed. For RNase R treatment, 2 μg total RNA was incubated with or without 6 U RNase R (Geneseed Biotech, Guangzhou, China, catalog no. R0301) for 30 min at 37°C. Subsequently, the expression of circTLCD4-RWDD3 and *TLCD4-RWDD3* mRNA were analyzed by qRT-PCR analysis.

For actinomycin D assays, 1 × 10^5^ per well of NSCLC cells were seeded in a 6-well plate before a treatment of 5 μg/ml actinomycin D (APExBIO, Houston, TX, USA, catalog no. A4448) at indicated time points (6, 12, 18 and 24 h). Then, total RNA was extracted from the harvested cells and analyzed using qRT-PCR analysis.

***Bioinformatics analysis***

The complex structure of circTLCD4-RWDD3 and hnRNPA2B1 was obtained from HDOCK structural alignment tool (http://hdock.phys.hust.edu.cn/). The binding motifs of hnRNPA2B1 were predicted by POSTAR3 (http://lulab.life.tsinghua.edu.cn/postar3/). The sequence analysis to predict the specific interacting site of circTLCD4-RWDD3 on *UBC9* or *PROX1* promoter were conducted using the plug-in Megalign in DNAstar software.
